# Drug Response Prediction as a Link Prediction Problem

**DOI:** 10.1038/srep40321

**Published:** 2017-01-09

**Authors:** Zachary Stanfield, Mustafa Coşkun, Mehmet Koyutürk

**Affiliations:** 1 Center for Proteomics and Bioinformatics, Case Western Reserve University, Cleveland, OH, 44106, USA; 2 Department of Electrical Engineering and Computer Science, Case School of Engineering, Case Western Reserve University, Cleveland, OH, 44106, USA.

## Abstract

Drug response prediction is a well-studied problem in which the molecular profile of a given sample is used to predict the effect of a given drug on that sample. Effective solutions to this problem hold the key for precision medicine. In cancer research, genomic data from cell lines are often utilized as features to develop machine learning models predictive of drug response. Molecular networks provide a functional context for the integration of genomic features, thereby resulting in robust and reproducible predictive models. However, inclusion of network data increases dimensionality and poses additional challenges for common machine learning tasks. To overcome these challenges, we here formulate drug response prediction as a link prediction problem. For this purpose, we represent drug response data for a large cohort of cell lines as a heterogeneous network. Using this network, we compute “network profiles” for cell lines and drugs. We then use the associations between these profiles to predict links between drugs and cell lines. Through leave-one-out cross validation and cross-classification on independent datasets, we show that this approach leads to accurate and reproducible classification of sensitive and resistant cell line-drug pairs, with 85% accuracy. We also examine the biological relevance of the network profiles.

The last decade has seen significant advances in high-throughput molecular profiling technologies^1^,^2^. As a result, precision medicine has gained much attention and extensive -omic datasets have been produced. This places bioinformatics at the forefront of personalized medicine research due to a need for techniques that can integrate and analyze such data in a way that can improve understanding of disease and potential treatment options^1^,^3^,^4^.

One such area of interest is the problem of drug response prediction. This involves using a patient’s molecular profile to estimate the effectiveness of a given drug on the patient. To address this problem, extensive patient drug screening projects need to be performed in order to discover significant patterns of response. However, it is not feasible to treat large populations of cancer patients with numerous drugs. To circumvent this issue in the context of cancer, two large drug screening projects have been carried out using cancer cell lines in place of patient samples. These are the Genomics of Drug Sensitivity in Cancer (GDSC) and the Cancer Cell Line Encyclopedia (CCLE) projects^5^,^6^. Both projects performed molecular profiling (somatic mutation, copy number variation (CNV), and gene expression screening) for hundreds of cancer cell lines and treated them with multiple established compounds. Data from these two projects are publicly available and have been studied extensively to develop and test methods of drug response prediction.

One of the largest works in drug response prediction, the NCI-DREAM Drug Sensitivity Prediction Challenge, obtained data on breast cancer cell lines and compiled 44 drug response prediction algorithms from participating researchers^7^. Through this work, the DREAM challenge brought the problem of drug sensitivity prediction to the forefront, stressing its relevance in the context of precision medicine. Furthermore, the challenge provided a basis on how to best approach this problem for the GDSC and CCLE datasets as well as existing patient data. Most techniques in the DREAM challenge built a feature set from the cell line molecular data for use in a machine learning algorithm. This entails learning drug models in which weights correspond to the importance of each feature for predicting cell line response, typically represented by IC50 values (the drug concentration required for 50% inhibition of cell growth). Following this procedure, another large drug prediction project, carried out by Jang *et al*., tested over 100,000 models based on well-known machine learning algorithms using combinations of molecular data from either the GDSC or CCLE^8^. This study showed that approaches employing gene expression data typically achieve higher performance with a majority of drugs reaching relatively good classification accuracy (AUC > 0.6).

While machine learning approaches have led to significant progress in drug response prediction, they also face important challenges in the context of this problem. The first is that of high dimensionality. Namely, the number of genes profiled is orders of magnitude larger than the number of samples, making feature selection difficult and vulnerable to overfitting. Second, since it is more difficult to learn from binary or discrete-valued features, effective use of mutation or copy number information in drug response prediction is challenging.

To overcome the challenges associated with applying machine learning to drug response prediction, network and pathway information can be utilized. The idea behind this approach is to provide a functional context for the features that are being used in classification, thereby improving the biological relevance, robustness, and reproducibility of resulting models. This has been done implicitly for certain machine learning algorithms via a modification to the training phase. The objective function optimized during training in order to learn feature weights for the final predictive model is altered by adding a network-constraint term^9^. This term biases the final feature weights such that highly connected nodes in the biological network are given similar weights. As for purely network-based predictive models, little work has been done in the context of drug response prediction. Zhang *et al*. constructed a dual-layered network consisting of cell lines and drugs where weighted edges between two nodes of the same type represented similarity and edges between cell lines and drugs contained the response data^10^. While the application of network-based approaches to drug response prediction is limited, network-based methods, specifically those employing link prediction, have been successful in many biomedical problems that involve predictive tasks. These problems include prediction of protein-protein interactions, drug-target interactions, and disease-gene associations^11^,^12^,^13^. Such results motivate the application of a similar network-guided prediction approach to the problem of predicting drug response.

In this work, we propose an alternate perspective to using network data in drug response prediction by formulating it as a link prediction problem. For this purpose, we integrate cell line mutation data and drug responses from the GDSC with a protein-protein interaction (PPI) network, creating a large, heterogeneous network consisting of genes, cell lines, and drugs. For each cell line and drug pair, we compute network profiles (representing the proximity of mutated genes in a cell line to every other node in the network) by performing a random walk with restart (RWR) on variations of the network. Network proximity utilizes functional links between proteins. Using this measure, we can smooth out the mutations to see how they may influence the overall biology of a cell. The “mutation profile” for a cell line provides information on the genes that are mutated in a given cell line. In contrast, the network profile computed by smoothing the mutation profile across the network provides information on the genes that are functionally associated with genes that are mutated in a given cell line. To this end, the incorporation of network proximity provides a functional context for the interpretation of mutations. We compute one network profile for each cell line and two network profiles for each drug, reduce the dimensionality of these profiles, and use these profiles to assess the likelihood of the existence of a “link” between each drug and cell line. Here, a link between a drug and cell line represents either the sensitivity or resistance of the cell line to the drug. In other words, based on the premise that different biological pathways may be underlying the sensitivity or resistance to a specific drug, we compute separate profiles for each drug that represent the functional bases of sensitivity or resistance to the drug. Subsequently, for each cell line-drug pair, we assess the similarity between the network-smoothed mutational landscape of the cell line and the resistance and sensitivity profiles of the drug to assess the likelihood of the sensitivity and resistance of the cell line to the drug. In this way, we score each cell line-drug pair, where the score is a prediction of sensitivity. We perform parameter analysis to investigate the value added by the use of the PPI network, and observe that the utilization of network information drastically improves prediction of response, as compared to mutational profiles alone. We also validate the resulting prediction method on the CCLE dataset to assess the ability of our method to predict response for unseen samples. Finally, we functionally annotate the sensitivity and resistance profiles of selected drugs to understand whether separate biological processes underlie sensitivity vs. resistance to a drug.

## Results

Our method uses existing heterogeneous datasets (drug screens, cell line mutations, and protein-protein interactions (PPIs)) to predict the effect of a drug on a given cell line. This is achieved by building a series of heterogeneous networks based on data from the Genomics of Drug Sensitivity in Cancer (GDSC) dataset. Given a new cell line, these networks serve as a model used to predict the response of this cell line to tested GDSC drugs. Similarly, given a new drug, these networks can be used as a model to score tested GDSC cell lines for their potential response to this drug.

**Figure 1:**
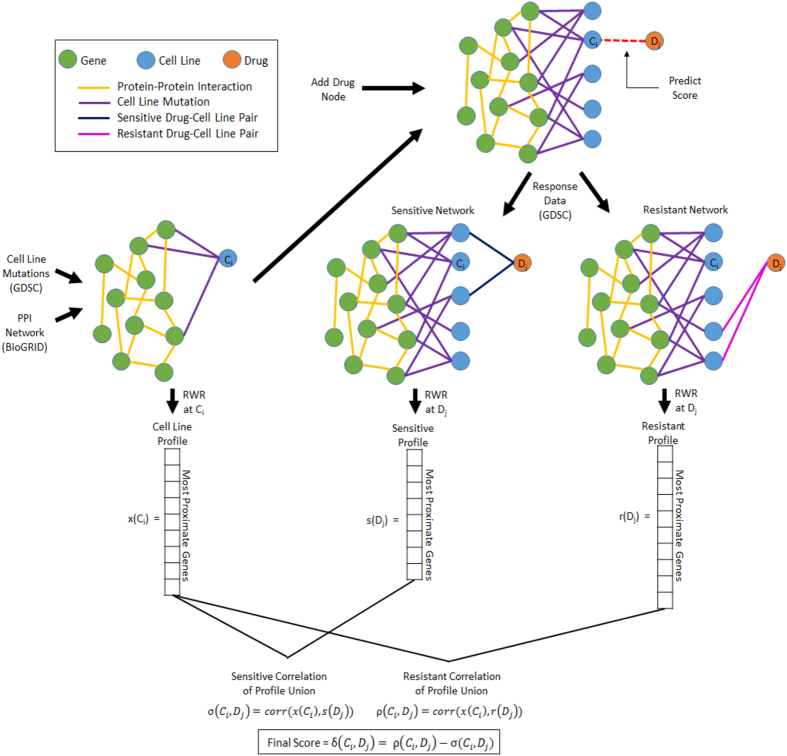
Workflow of the proposed computational method for network-based prediction of drug response. Network profiles for cell lines and drugs, vectors representing proximity to genes mutated in cell lines of interest, are generated at separate stages using a fast random walk with restart (RWR) on a heterogeneous network consisting of edges representing response of cell lines to drugs, mutations of genes in these cell lines, and interactions among proteins coded by these genes. Two profiles are calculated for a drug, sensitivity profile and resistance profile by using networks that respectively contain sensitive cell lines and resistant cell lines separately for each drug. Based on the similarity of these profiles, a sensitivity score and a resistance score is calculated for each cell line-drug pair. The final score for each cell line-drug pair is obtained by subtracting the sensitivity score from the resistance score. The final score is used to assess the likelihood that the cell line is sensitive to the drug.

The workflow of the proposed method is shown in Fig. 1, and described in detail in the Methods Section. For existing cell line and drug data, the method first constructs network profiles for drugs and cell lines, representing genes that are functionally related to genes mutated in the respective cell line (or cell lines responsive/resistant to the respective drugs). For each drug, two network profiles are constructed, one representing the cell lines that are sensitive to the drug, one representing those that are resistant. Subsequently, to predict the potential response of a given cell line to a given drug, we assess the similarity between the respective network profiles. Based on the correlations between network profiles, we obtain two scores for each cell line-drug pair: a “sensitivity score” and a “resistance score”. The sensitivity score represents the correlation between the network profile of the cell line and the sensitivity profile of the drug, whereas the resistance score represents the correlation between the network profile of the cell line and the resistance profile of the drug. To assess the likelihood that the cell line is sensitive to the drug, we use the difference between the sensitivity score and resistance score.

Our approach is based on the premise that a cell line that is sensitive (resistant) to a given drug will have a network profile that is more similar to the network profiles of other cell lines that are also sensitive (resistant) to the drug, as compared to those that are resistant (sensitive). This hypothesis is grounded in the notion that protein-protein interaction networks provide a functional context for molecular perturbations, including cancer mutations. Indeed, integration of mutations with protein interactions has been used to identify sets of genes important for different cancers and tumorigenic pathways^14^,^15^,^16^. Furthermore, these network-based approaches have successfully used mutation data to stratify patients into different groups based on survival or cancer subtype^17^,^18^. These results demonstrate that introducing functional associations to mutations provides insight into dysregulated networks in cancer. Since such dysregulated networks are associated with different cancer subtypes or patient survival, they potentially contain information on drug response as well. In other words, linking cell lines to drugs based on sensitivity provides a functional context for the relationship between the mutations (and their corresponding dysregulated networks) and drug response.

### Leave-One-Out Cross-Validation on the GDSC Dataset

In order to evaluate the accuracy of our method, we perform leave-one-out cross validation (LOOCV) using the GDSC data. For every cell line-drug pair, the corresponding nodes in the network are connected by exactly one edge, either in the sensitivity or resistance network for the respective drug. By removing this edge during profile calculation, we create a cell line-drug pair for testing. We then use the remaining GDSC data, via the network, to score this pair. This effectively reduces the problem to link prediction between the nodes representing the cell line and drug pair. To comprehensively assess the prediction performance of the proposed method, we repeat this process for all cell line-drug pairs in the dataset.

**Figure 2:**
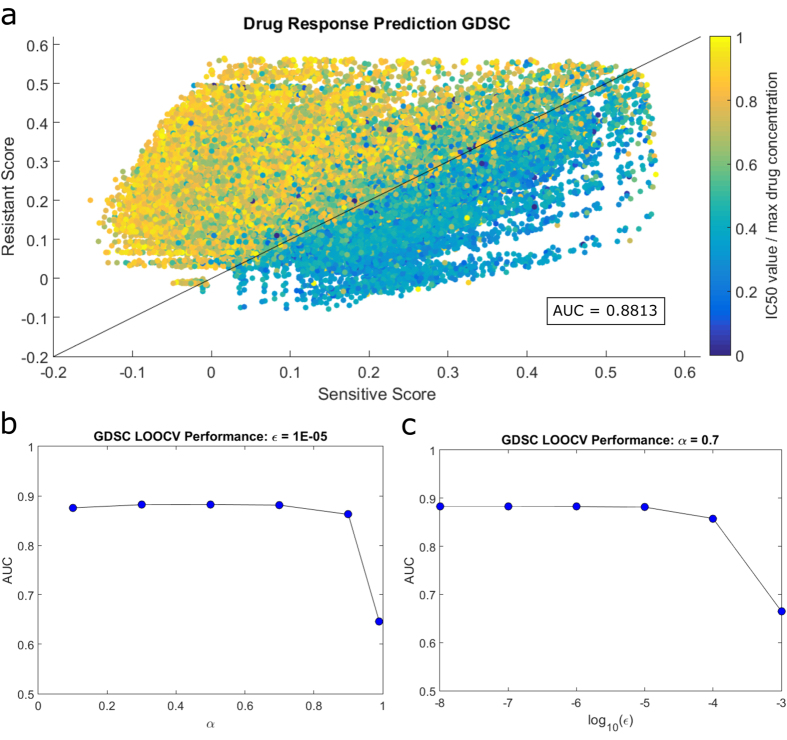
Performance of the proposed link prediction method on the GDSC Dataset. (**a**) Each data point on the graph corresponds to a single cell line-drug pair. The x axis shows the predicted sensitivity score, the y axis shows the predicted resistance score for each cell line-drug pair. The color reflects the measured IC50 value for that pair: yellow indicates resistant and blue indicates sensitive. The black line (*y* = *x*) is the intuitive classification line of sensitive and resistant pairs as any data point below the line will have a higher sensitivity score than resistance (and vice versa). (**b**) Prediction performance as a function of the restart probability in random walk with restarts. (**c**) Prediction performance as a function of the parameter (*ε*) that controls the sparsity of the network profiles. The left-most point corresponds to using the full profiles (i.e. utilization of all genes in the network profiles). (**b**) and (**c**) show that when the network information used is very limited, performance decreases. For **a**, the following parameters were used: *α* = 0.7, *ε* = 1*e* − 5.

We compare the scores for each test pair (686 cell lines and 138 drugs, leading to 94,668 total pairs) with normalized IC50 values from the GDSC drug screening data to evaluate classification performance for different values of parameters (restart probability, threshold for dimensionality reduction). We use area under the receiver operating characteristic curve (AUC) to measure accuracy. The two response classes for cell line-drug pairs are sensitive and resistant, which are assigned to pairs with IC50 values respectively less than or greater than the maximum used concentration of the drug in the screening assay. Overall, we observe that the prediction performance of the method is quite robust to the selection of parameters, and there is a combination of parameters that leads to an AUC of 0.8813. To visualize how cell line-drug pairs are scored, we plot the relationship between predicted sensitivity scores, predicted resistance scores, and normalized IC50 values for each cell line-drug pair (Fig. 2a). In this figure, each pair is represented by a single point colored by the IC50 value of the respective pair in the GDSC response data. In other words, the values on the x-axis (sensitivity score) and y-axis (resistance score) are computed with no knowledge of color (the reported IC50 value) for the drug-cell line pair; only the colors (IC50 values) of other points (drug-cell line pairs) are used in computing these scores (leave-one-out-cross validation). Yet, as seen in Fig. 2a, with only two features, namely resistance and sensitivity scores, clear separation of sensitive and resistant pairs is achieved.

### Effect of Parameters on Prediction Performance

During the calculation of sensitivity and resistance scores, we use two parameters. These are the restart probability in the RWR, *α* (used to bias the random walk toward genes mutated in cell lines of interest), and the profile sparsity threshold, *ε* (used to reduce the dimensionality of the profiles, but restricting focus on only the genes that are strongly functionally associated with genes mutated in cell lines of interest). In order to understand the affect of *α* and *ε* on prediction accuracy, we perform LOOCV analysis with varying values of each parameter (Fig. 2b and c). A larger *α* restricts the random walker to the mutated genes’ neighborhood as the walker is less likely to travel far from its starting position. For values of *α* that are close to 1, we essentially only allow the cell line mutations to be scored highly in the profile. A smaller *α* produces a more smooth score distribution because the network is able to have a substantial effect on when each nodes is visited by the random walker. Therefore, as the value of *α* increases, the influence of the network becomes more limited. The other parameter, *ε*, allows us to set a cutoff for how highly a gene needs to be scored in the profile to be considered relevant in relation to response. This parameter reduces dimensionality and helps reduce noise as taking the correlation of very large vectors could lead to a background effect of the network that may limit the variation across pair scores.

In the LOOCV, values of *α* ranging from 0.1 to 0.7 result in highly similar performance, while very large values produce a sharp decrease in performance (Fig. 2b). We observe a similar trend for the effect of *ε*, with quite stable performance until *ε* is relatively large (Fig. 2c). These results suggest that restricting the profiles to mutations only, either by limiting the network influence or the number of genes in the profiles, results in a decline in prediction performance. In other words, increasing network influence or profile size (by decreasing *α* and/or *ε*) improves classification accuracy to a certain degree. This implies that somatic mutations alone may not be strong predictors of drug response, but significant value is added when they are incorporated into a network of functional associations. Finally, the prediction performance for large *α* and *ε* is similar to that provided by machine learning approaches that utilize only mutation data, which again demonstrates the value of the information added by the PPI network.

### Cross-Classification Performance

**Figure 3:**
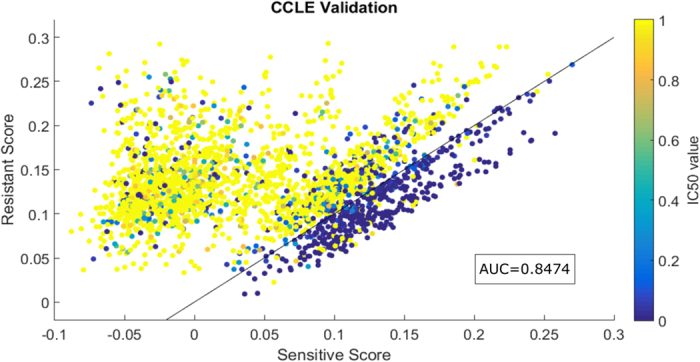
Performance of the proposed link prediction method “trained” using cell lines from GDSC on cell lines obtained from CCLE. As in Fig. 2a, each data point is a cell line-drug pair where the x-axis and y-axis respectively show the predicted sensitivity and resistance scores. The colors here corresponds to the IC50 values in the CCLE dataset for the 2,585 new pairs. The following parameters values were used: *α* = 0.7, *ε* = 1*e* − 5.

To simulate making predictions for an unseen sample, we evaluated our method using the CCLE dataset. For each of the 235 cell lines obtained from CCLE (i.e. not included in the GDSC project) a representative node and mutation edges are added to the existing GDSC-based network. A final prediction score is then calculated for each of the 11 drugs shared by the CCLE and GDSC datasets. We then evaluated these prediction scores by using the IC50 values in the CCLE response data. In total, 2,585 new cell line-drug pairs are scored. Similar to our observation for the LOOCV analysis, we observe that sensitive and resistant pairs are separated well by the two correlation scores for the cross-classification analysis as well, leading to an AUC of 0.8474 (Fig. 3). This result helps demonstrate the robustness and reproducibility of our network-based approach.

### Individual Cell Linle and Drug Performance

As discussed in the previous section, the method proposed is highly accurate in both a LOOCV (within GDSC) and a cross-classification (training on GDSC, testing on CCLE) framework in terms of classifying sensitive and resistant cell line drug pairs. In practice, the problem can be posed in two different settings: (i) predicting effective drugs for a given cell line vs. (ii) predicting resistant cell lines for a given drug. The former setting is more likely to be relevant in a clinical setting, in that the clinician may be interested in identifying drugs that are likely to lead to better outcomes for a given patient. The latter setting, on the other hand, can be useful in research and drug development, in which researchers identify (additional) cancers that can be targeted for a new or an existing drug. In this section, we assess and compare the performance of the proposed method in these two settings, with a view to characterizing the practical applicability of the method, as well as gaining insights into the behavior of our method.

**Figure 4:**
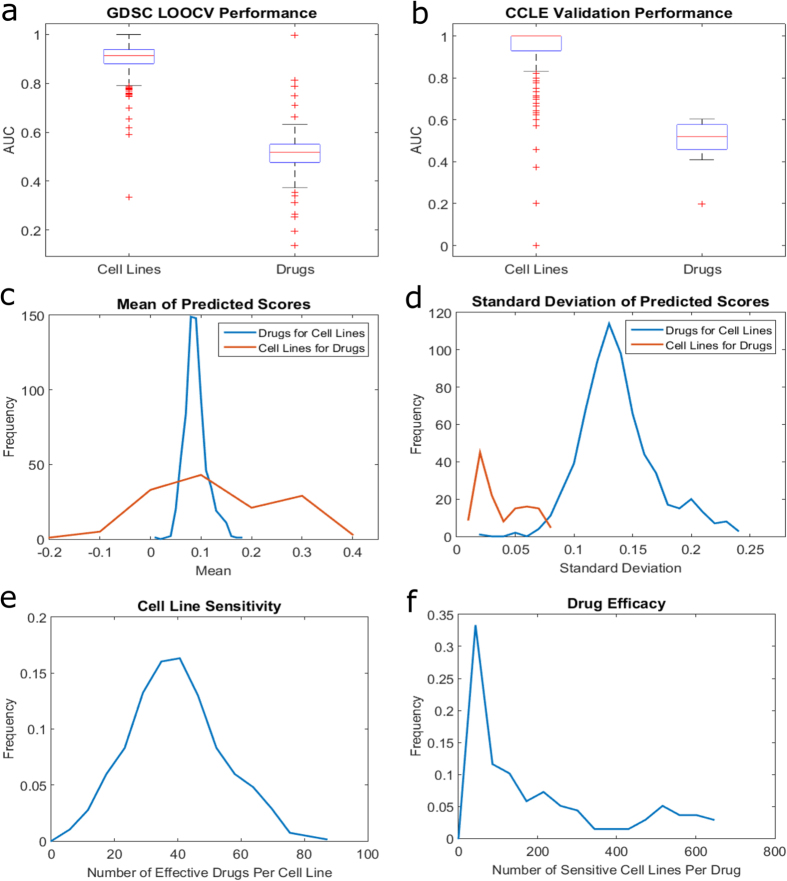
Performance of network-based classification for predicting “drugs for cell lines” vs. “cell lines for drugs”. The area under ROC curve (AUC) for leave-out-cross validation (LOOCV) on GDSC and cross-classification on CCLE based on GDSC-training for the two different settings are shown respectively in (**a**) and (**b**). “Cell lines” refers to predicting drugs for a given cell line, whereas “Drugs” refers to predicting cell lines for a given drug. The distribution of means and standard deviations of predicted scores for drugs (cell lines) per cell line (drug) in the GDSC LOOCV experiments are shown respectively in (**c**) and (**d**). The distribution of cell line sensitivity across drugs and drug efficacy across cell lines in the GDSC data are shown respectively in (**e**) and (**f**).

For this purpose, we comprehensively compute performance figures for all cell lines in both the GDSC LOOCV and CCLE validation (Supplementary Tables S1 and S3). Similarly, we arranged scores in a per drug manner to understand how well our method classifies the set of cell lines for a given drug (Supplementary Tables S2 and S4). Organizing the scores by cell line provides insight into how well our method can separate out effective and ineffective therapies for a new cell line (or patient in a translational setting). Arranging the scores by drug shows how accurately responsive and non-responsive cell lines can be separated for a given drug. The results of this analysis using area under ROC curve (AUC) as the performance criterion are shown in Fig. 4a and b (please see Supplementary Tables S1–S4 for other performance criteria). As seen in the figure, our method very accurately distinguishes effective drugs from non-effective ones for each cell line. However, this method performs only slightly better than random, on average, for the task of separating sensitive and resistant cell lines for each drug. This observation suggests that the method presented here is better suited to the task of predicting effective drugs for a given cell line.

To understand the underlying reasons for the performance gap between the two different settings, we examine the characteristics of the predicted scores in the context of LOOCV on GDSC. The results of this analysis are shown in Fig. 4c and d. As seen by the blue curves in Fig. 4c and d, predicted scores for drugs per cell line have a similar distribution across all cell lines, showing a quite consistent mean score with a high variance over all scores. In contrast, as seen by the orange curves in Fig. 4c and d, we observe a wide range of mean predicted scores and a small variance among cell line scores per drug. Based on the mean and the standard deviation of score distributions per cell line and per drug, low drug performance is largely explained statistically (Fig. 4c and d). This observation is consistent with the performance gap between the two settings. Namely, the score distributions for cell lines allows the overall classification of individual cell line-drug pairs to be highly precise, while the small variance among cell line scores per drug make it difficult to accurately classify these cell lines. The difference between the distributions of predicted scores in the two settings can be explained by the distribution of sensitivity for cell lines and drugs. Cell lines have a near-normal distribution for the number of drugs to which they are sensitive, with the mean being around 40 drugs, about 29% (Fig. 4e). Drugs have a highly skewed distribution with most being effective against less than 40 cell lines, average of 5.25% (Fig. 4f). Together with the low variance of cell line scores, this large difference among classes lowers drug performance. Another possible reason for the performance gap could be the way the network is structured. Cell line nodes carry more importance as they link the PPI to drugs and have more connections. Drug profiles created from resistance networks (i.e. linking drugs to resistant cell lines) are likely to be very similar based on Fig. 4f (most drugs are ineffective against a large majority of cell lines), while the opposite is true for drug profiles from sensitivity networks. This is also likely to play a role in the accuracy and score distributions seen in Fig. 4a–d. These observations suggest that the performance of network-based prediction of sensitive cell lines for a given drug can be improved by comprehensively characterizing the molecular effects of drugs and adding links to the network to represent these effects.

### Method Comparison

**Figure 5:**
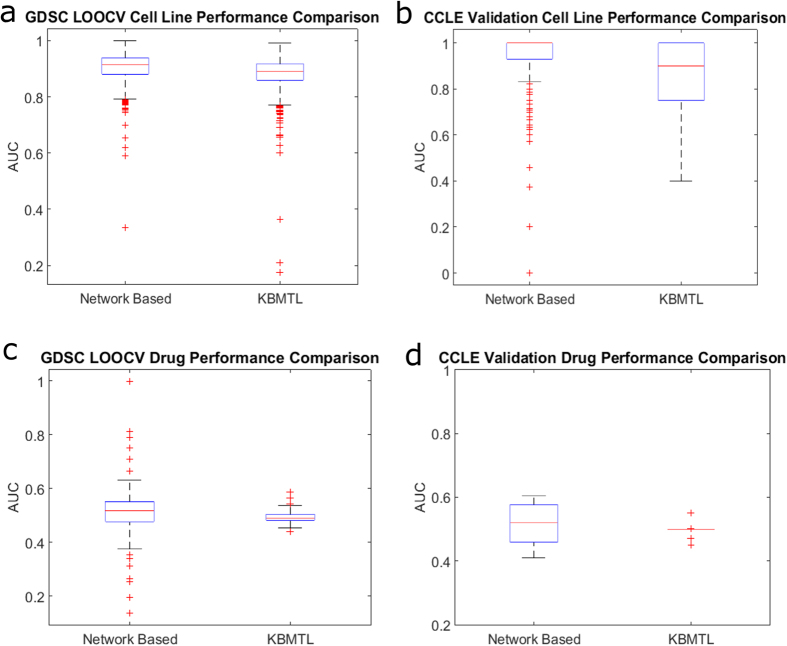
Comparison of the performance of the proposed network-based prediction algorithm against the KBMTL algorithm^19^. The distribution of the area under ROC curve achieved by each algorithm for predicting drugs for each cell line is shown for LOOCV on GDSC (**a**) and cross-classification on CCLE with training on GDSC (**b**). The distribution of the area under ROC curve achieved by each algorithm for predicting cell lines for each drug is shown for LOOCV on GDSC (**c**) and cross-classification on CCLE with training on GDSC (**d**).

In order to better understand the accuracy of our method, we compare it against the top performing approach in the DREAM Drug Sensitivity Prediction Challenge, Gonen and Margolin’s kernelized Bayesian multitask learning (KBMTL) algorithm^19^. This approach attempts to predict the susceptibility of each cell line to a panel of drugs simultaneously, which is comparable with our method and goal of ranking drugs for each cell line. We apply the binary classification implementation of the KBMTL algorithm to both the GDSC and CCLE datasets using default parameters and 100 training iterations, with features being cell line mutations (binary) and IC50 value as the response variable. Figure 5a and b show the performance comparison via the distribution of AUCs for cell lines and drugs, respectively, between our network-based method for GDSC LOOCV and 50 runs of 10-fold cross validation of KBMTL on the GDSC dataset. The mean cell line and drug AUCs for the method presented in this work are 0.9038 ± 0.06 and 0.5147 ± 0.10, while KBTML achieves average AUCs of 0.8813 ± 0.07 and 0.4938 ± 0.02. Performing a paired t-test between these AUC distributions results in p-values of 2.893*e* − 11 for cell lines and 0.0165 for drugs. The equivalent comparison is performed for CCLE validation (i.e. training on all GDSC cell lines, testing on the 235 cell lines not in the GDSC, and assessing accuracy using the CCLE IC50 values). Figure 5c and d show distributions of AUC values for cell lines and drugs, respectively. The network-based method presented in this work achieves an average AUC of 0.9391 ± 0.12 for cell lines and 0.4958 ± 0.12 for drugs. For KBMTL, these are 0.8763 ± 0.13 and 0.4973 ± 0.02. A paired t-test results in p-values of 1.834*e* − 07 for cell lines and 0.967 for drugs. Together, these results show that in a cross validation problem, our method significantly outperforms the KBMTL algorithm for both prediction tasks (classifying drugs for cell lines and classifying cell lines for drugs). Additionally, when making predictions for new samples (cell lines), which is the clinically relevant setting, we significantly outperform KBMTL.

### Functional Annotation of Drug Profiles

**Figure 6:**
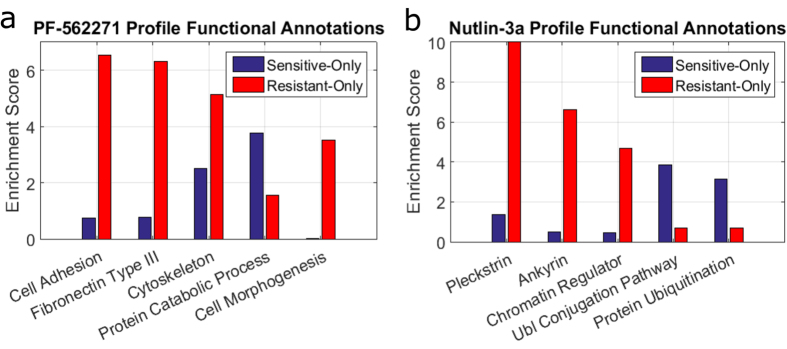
Functional annotation of drug profiles. Genes included in sensitivity and resistance profiles for two drugs are input into the online tool DAVID for functional enrichment analysis. Genes occurring in both profiles are excluded from this analysis. A subset of the most enriched annotations are chosen for comparison for two drugs, PF-562271 (**a**) and Nutlin-3a (**b**). PF-562271 targets focal adhesion kinase, which is involved in cellular adhesion. Four terms relating to adhesion are highly enriched in the resistant node set versus the sensitive. Conversely, ubl conjugation pathway and protein ubiquitination are highly enriched in the sensitive node set for Nutlin-3a, whose target is Mdm2 (or E3 ubiquitin-protein ligase).

Cross-validation and cross-classification results show that our profile-based scoring method allows us to accurately predict drug response. A natural question that arises from this is what additional information is being provided by these network profiles, i.e. are the genes added to a cell line’s profile by the network biologically relevant in terms of response? To seek an answer to this question, we functionally annotate genes occurring in the sensitivity and resistance profiles of two drugs, PF-562271 and Nutlin 3a, using the web-based functional enrichment tool DAVID^20^. We chose these drugs due to the specificity of their targets, focal adhesion kinase (FAK) for PF-562271 and Mdm2 (E3 ubiquitin-protein ligase) binding with p53 for Nutlin 3a. This characteristic should allow easier interpretation of the profile annotations over chemotherapeutics or broad kinase inhibitors. For both drugs, we perform functional enrichment analysis on genes occurring exclusively in the sensitivity/resistance profile for that drug for *ε* score threshold of 1e − 5 and restart probability of 0.7. PF-562271 inhibits FAK and terms relating to the function of FAK have much higher enrichment in the genes that are recruited into the resistance profile (Fig. 6a). On the other hand, for Nutlin 3a, the sensitive-only gene list has a substantially higher enrichment for target-related terms, ubl (ubiquitin ligase) conjugation pathway and protein ubiquitination, relative to the genes that are exclusive to the resistance profile (Fig. 6b). Together, these two results suggest that the enrichment of terms related to the function of a drug’s target are significantly different between the sensitivity and resistance profiles. The profile showing the high enrichment may differ between drugs, but this large differential enrichment indicates functional relevance of the profiles and may partly explain our accuracy of classification.

## Discussion

The method employed in this work is, to our knowledge, the first drug response prediction method to directly integrate response data, cell line mutation data, and PPIs into a network. There are previous approaches that only use a subset of such data or build heterogeneous networks, which generally link nodes of the same type by similarity, such as gene expression for cell lines or chemical structure for drugs^10^,^12^. Here we employ the GDSC dataset as a knowledge-base upon which new predictions can be made. The result is highly accurate response classification. Additionally, our method classifies cell line-drug pairs individually. This offers multiple opportunities for analysis, such as finding the most effective drug for a patient, the most sensitive cell line for a drug, and the most responsive cell line-drug combinations.

Another important outcome of this study is that during the LOOCV, our method calculates a score for each missing pair, however, almost 20% of the IC50 values from the GDSC dataset are missing. Therefore, these predictions can be used to provide estimates for the missing IC50 values, which may direct future research. The scores we can provide may be used to determine which cell line-drug combinations would be most or least effective. If a researcher wants to find which drugs will induce strong response from their cell line of interest, our estimates could suggest other drugs, not tested against this cell line by the GDSC project, that may be more effective. Similarly, research projects focusing on drug resistance could make use of our response scores by testing pairs predicted to be ineffective. Compared to screening all missing combinations, this would reduce the time and resources required to answer numerous research questions.

Predictive model building for drug response provides the ability to interpret what genes or pathways contribute to sensitivity for cell lines or effectiveness for drugs. As for the method applied in this work, this information is contained in the network profiles. Comparing the network profiles for effective drugs with ineffective ones for a single cell line may provide insight into what genes or pathways need to be influenced by a drug for this specific cell line to respond. Similarly, using the profiles of sensitive and resistant cell lines, it may be possible to determine which pathways allow a specific drug to be effective or ineffective.

This network-based method utilizes cell line somatic mutations. However, it is possible that the accuracy of this method could be improved by incorporating additional data types (expression and copy number variations (CNVs)). In doing so, network profiles of cell lines and drugs could then be compared for different data used. Such comparisons may show what information each data type adds and how it influences response prediction. Similarly, optimal parameter values may be different between the types of data used, which could provide insights into the influence or importance of the network for each data type.

It is important to note that the set of real functional connections in a given cancer cell line will be different from the PPI network used here, since the latter includes connections that have been validated in any tissue or cell type. For this reason, the effectiveness of the methods proposed here can be improved by using networks consisting of interactions that are specific to each cell line, such as tissue-specific networks that are inferred by using tissue-specific gene expression data^21^,^22^. Indeed, it was shown that utilization of tissue-specific networks enhances discovery and prioritization of genes associated with many complex diseases, including cancer^23^. However, it is not computationally straightforward to adopt the proposed computational framework to utilize cell line or tissue-specific networks, since incorporation of cell line or tissue specificity requires use of a different network for each cell line. Therefore, development of methods to effectively utilize cell line or tissue specific networks would be an essential next step in network-based prediction of drug response.

In cases such as cancer, the same disease may present quite differently in a group of patients according to their molecular profiles. For this reason, the best therapy for two individuals diagnosed with the same disease may not be equivalent. By utilizing patient molecular data, we can link these biological differences among patients to make accurate and personalized choices for drug treatment. Following this idea, our method employs a functional association network with a drug screening dataset to construct an accurate model for predicting drug response for a sample/patient with a given set of genetic mutations. This model could be extended to other applications in biology, such as disease-drug prediction and drug-target prediction, while the sparse network profile correlation procedure can be a valuable contribution to works attempting to score proximity/similarity of nodes in a network. Network-based prediction methods can incorporate large amounts of existing data while offering an alternative way of answering biological problems. In this work, we were able to explicitly show the value of the PPI network and improve upon previous approaches in the area of drug response prediction.

## Methods

### Data

Cell line response data was downloaded from the Genomics of Drug Sensitivity in Cancer (GDSC) project website. This data includes log-normalized IC50 values for 707 cancer cell lines treated by 140 drugs. As suggested in the GDSC documentation, IC50 values were further normalized by the maximum drug concentration used in the screening assay. Cell lines with an IC50 value less than the maximum applied concentration of a drug were labeled as sensitive and the rest (those whose IC50 is greater than the maximum concentration) as resistant.

Cell line mutations were obtained from COSMIC^24^. Only mutations predicted to be pathogenic by the tool Functional Analysis through Hidden Markov Models (FATHMM) were used in the analysis, reducing the number of mutations to 315,214 out of the original total of 654,445^25^. The FATHMM predictions were obtained by COSMIC and are included in the COSMIC mutation dataset.

Cell lines used in this work were limited to those with mutation information and those showing sensitivity to a least one drug. Similarly, drugs were not considered if they were not effective against at least one cell line. Application of these filters resulted in a final set of 686 cell lines, with 170,164 mutations, and 138 drugs.

Finally, the protein-protein interactions included in the network were obtained from BioGRID^26^. This PPI network contains 19,165 proteins and 145,440 interactions.

### Computing Network Profiles for Cell Lines and Drugs

We represent the PPI network as *G* = (*V, E*), where *V* denotes the set of vertices (proteins) and *E* denotes the set of edges (interactions). To calculate the network profile of a given cell line *c*_*i*_, denoted *x(c*_*i*_) in Fig. 1, a node representing the cell line is first added to the PPI network. For each gene *g*_*k*_ that is mutated in *c*_*i*_, an edge with unit weight is added between the node representing *c*_*i*_ and the node representing the protein coded by *g*_*k*_. Ideally, edges linking cell lines to mutations would be weighted based on a score that represents the mutation’s importance to the phenotype (i.e. driver vs passenger mutations)^27^. However, we are not able to obtain comprehensive and consistent scores for all cell lines and mutations using existing tools. For this reason, in our experimental studies, we do not assign weights to the edges between cell lines and mutations. However, it is straightforward to incorporate mutation scores into the proposed framework as they become more comprehensive and reliable. The network can now be written as 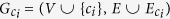, where 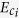 is the set of edges between *c*_*i*_ and the products of genes that are mutated in *c*_*i*_. The profile of *c*_*i*_ is then computed by performing a random walk with restarts (RWR) on 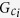, with restart vector seeded at the node representing *c*_*i*_. RWR is a network proximity measure that is defined as follows:


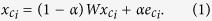


Here, *W* denotes the stochastic matrix derived from the adjacency matrix of the network, 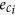 denotes the restart vector that contains a 1 for *c*_*i*_ and zeros for all other nodes, and *α* denotes the restart probability (also known as the damping factor). The restart probability parameter configures the relative importance of the network context vs. identity of individual mutations in a cell line, where a larger *α* weighs individual mutations more than network proximity to mutations. As presented in the Results section, we comprehensively assess the effect of this parameter on prediction performance. Defined this way, 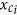 is a vector in the space induced by the nodes in the network and contains values in the interval [0, 1] at each dimension (i.e. for each node). These values represent the proximity to the seed node, in this case the cell line. The network profiles can be computed iteratively, however, the computation can be costly since it is repeated for all cell lines and twice for all drugs (as explained below). For this reason, we here use Chopper a fast Chebyshev-polynomial based algorithm for computing RWR-based proximity^28^.

To compute network profiles for drugs, we add all cell line nodes and corresponding mutation edges to the network to obtain a Protein-Cell Line-Mutation (PCM) network. We represent this network as *H* = (*V* ∪ *C, E* ∪ *E*_*C*_), where *C* is the set of all cell lines and *E*_*C*_ is the set of edges representing mutations for all cell lines, i.e. 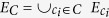. Using this integrated network, we compute two network profiles for each drug: a “sensitivity profile” and a “resistance profile”. To compute the sensitivity profile *s(d*_*j*_) for drug *d*_*j*_, we add to the PCM network edges connecting *d*_*j*_ to the cell lines that are sensitive to *d*_*j*_ and assign weights to these edges representing the corresponding normalized IC50 values. In other words, the sensitivity network for drug *d*_*j*_ is defined as 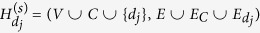, where 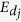 is the set of edges connecting *d*_*j*_ to cell lines that are sensitive to *d*_*j*_. We then compute *s(d*_*j*_) by performing a RWR on 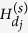, with restart vector seeded at the node representing *d*_*j*_. Similarly, to compute the resistance profile *r(d*_*j*_) for drug *d*_*j*_, we add to the PCM network edges connecting *d*_*j*_ to the cell lines that are resistant to *d*_*j*_, and assign weights to these edges representing the corresponding normalized IC50 values. The resulting resistance network for *d*_*j*_ is denoted 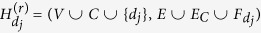, where 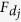 consists of the edges connecting *d*_*j*_ to cell lines resistant to *d*_*j*_. We then compute *r(d*_*j*_) by performing RWR on 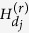, with restart vector seeded at the node representing *d*_*j*_.

### Scoring of Cell Line-Drug Pairs

Our objective is to compute a prediction score for the sensitivity of a given cell line to a given drug based on the network profiles computed as described in the previous section. For this purpose we use the similarities between the network profiles of cell lines and the sensitivity/resistance profiles of drugs. To measure the similarity between cell line and drug profiles, we employ the Pearson correlation coefficient. Namely, for a given cell line and drug pair, denoted respectively as *c*_*i*_ and *d*_*j*_, we compute a sensitivity score, *σ*_*ij*_ = *corr(s(d*_*j*_), *x(c*_*i*_)), and a resistance score, *ρ*_*ij*_ = *corr(r(d*_*j*_), *x(c*_*i*_)). Since these vectors are very high-dimensional (each dimension represents a node in the network and there are tens of thousands of nodes in the network) and sparse (the entry for a majority of nodes in the vector is negligibly small), we perform dimension-reduction before computing the correlations. For this purpose, we use a sparsity (or dimension reduction) parameter *ε*. Namely, while computing the correlation between *s(d*_*j*_) and *x(c*_*i*_), we remove any gene with a proximity score less than *ε* in both *s(d*_*j*_) and *x(c*_*i*_) from both profiles. A final prediction score, *δ*, is then calculated for a cell line-drug pair by subtracting the sparse sensitivity correlation from the sparse resistance correlation, or *δ*_*ij*_ = *ρ*_*ij*_ − *σ*_*ij*_. This prediction score is designed to be proportional to the maximum concentration-normalized IC50 values. A cell line that is sensitive to a drug is expected to have a higher correlation with that drug’s sensitivity profile than its resistance profile, making *δ*_*ij*_ < 0. Correspondingly, a cell line sensitive to a drug will have an IC50 value less than zero after normalization by the drug’s maximum concentration used in the assay and then by log-normalization (IC50 values are log-normalized drug concentrations). Therefore, our prediction score *δ*_*ij*_ and the real response value are negative for sensitive cell line-drug pairs and positive for resistant pairs.

It is important to note that during the GDSC leave-one-out cross validation, if there is an edge between the cell line and drug nodes of the current pair being tested, it is removed before the drug profile calculations. This ensures that the prediction on the test pair is done without the pair’s response data. During validation with other datasets, this is not an issue as the scores are calculated for new drugs or cell lines, meaning there cannot be an edge in the network connecting the pair.

### CCLE Validation

CCLE drug response and cell line mutation data was downloaded from the Broad Institute website. Cell lines not present in the GDSC project were scored against the drugs shared between both datasets using the GDSC-based network. For each new cell line, a corresponding node and mutation edges were added to the network for calculation of cell line and drug profiles. A score was obtained for all combinations between the 235 CCLE-only cell lines and the 11 drugs shared by CCLE and GDSC. These scores were then compared to the reported IC50 values in the CCLE dataset to determine prediction accuracy.

### Profile Functional Annotation

From the two profiles of a selected drug, node sets representing sensitivity and resistance were generated by extracting genes having RWR scores greater than the *ε* parameter. Genes shared between these two sets were removed to produce sensitive-only and resistant-only sets of proximal genes per drug. These were individually used as input lists for the web-based tool DAVID for functional enrichment^20^. The most significantly enriched annotations and their enrichment scores were compared between the two sets and with known drug action information.

## Additional Information

**How to cite this article:** Stanfield, Z. *et al*. Drug Response Prediction as a Link Prediction Problem. *Sci. Rep.* **7**, 40321; doi: 10.1038/srep40321 (2017).

**Publisher's note:** Springer Nature remains neutral with regard to jurisdictional claims in published maps and institutional affiliations.

## Supplementary Material

Supplementary Tables
