# Corrigendum: Drug Response Prediction as a Link Prediction Problem

**DOI:** 10.1038/srep44961

**Published:** 2017-03-23

**Authors:** Zachary Stanfield, Mustafa Coşkun, Mehmet Koyutürk

Scientific Reports 7: Article number: 4032110.1038/srep40321; published online: 01 09 2017; updated: 03 23 2017

This Article contains a typographical error in the Results section under the subheading ‘Method Comparison’.

“In order to better understand the accuracy of our method, we compare it against the top performing approach in the DREAM Drug Sensitivity Prediction Challenge, Gonen and Margolin’s kernelized Bayesian multitask learning (KBMTL) algorithm^19^”.

should read:

“In order to better understand the accuracy of our method, we compare it against a state-of-the-art approach, Gonen and Margolin’s kernelized Bayesian multitask learning (KBMTL) algorithm^19^”.

