# Supplementary material for: Drug Response Prediction as a Link Prediction Problem
Source: Sci Rep. 2017 Jan 9;7:40321. doi: 10.1038/srep40321 (PMC5220354; doi:10.1038/srep40321)
Supplement: Supplementary Tables [file srep40321-s1.pdf]

Drug Response Prediction as a Link Prediction Problem

Zachary Stanfield, Mustafa Coşkun, and Mehmet Koyutürk

Supplementary Table 1

Cell Line	Correlation	C-Index	AUC	Accuracy	Sensitivity	Specificity
MC-CAR	0.7645	0.7837	0.9338	0.8551	0.7234	0.9231
PFSK-1	0.7205	0.7806	0.9621	0.8980	0.9615	0.8750
A673	0.7000	0.7782	0.9541	0.8673	0.8750	0.8636
ES3	0.6594	0.7328	0.8953	0.8188	0.6591	0.8936
ES5	0.5962	0.7182	0.8777	0.8188	0.6667	0.8854
ES7	0.6056	0.7032	0.8629	0.8333	0.7045	0.8936
EW-11	0.5824	0.6877	0.8948	0.8406	0.7419	0.8692
NCI-H1395	0.4634	0.6462	0.7797	0.6739	0.7692	0.6640
COLO-829	0.5213	0.6731	0.8351	0.7556	0.6316	0.8041
5637	0.7804	0.8024	0.9256	0.8469	0.7027	0.9344
RT4	0.5600	0.7163	0.8804	0.8041	0.7857	0.8072
SW780	0.5114	0.6712	0.8035	0.7143	0.5313	0.8030
TCCSUP	0.7595	0.7869	0.9225	0.8367	0.7931	0.8551
C-33-A	0.7355	0.7786	0.9430	0.8866	0.7941	0.9365
ME-180	0.7754	0.7987	0.9610	0.8980	0.7500	0.9839
8-MG-BA	0.7896	0.8088	0.9023	0.8261	0.6491	0.9506
A172	0.7382	0.7972	0.9436	0.8764	0.7317	1.0000
GB-1	0.7589	0.7809	0.9061	0.8261	0.6296	0.9524
T98G	0.6877	0.7557	0.9250	0.8571	0.7105	0.9500
U-118-MG	0.7344	0.7705	0.8407	0.7755	0.6667	0.8108
U-87-MG	0.6981	0.7539	0.9394	0.8686	0.9130	0.8596
YKG-1	0.8146	0.8332	0.9637	0.8061	0.5957	1.0000
ChaGo-K-1	0.7341	0.7775	0.9565	0.8866	0.9130	0.8784
NCI-H720	0.4494	0.6385	0.7922	0.7464	0.5600	0.7876
Calu-3	0.7662	0.7799	0.9153	0.8265	0.7500	0.8571
COR-L23	0.8212	0.8273	0.9628	0.8163	0.6304	0.9808
LK-2	0.7242	0.7713	0.9292	0.8673	0.8065	0.8955
NCI-H1437	0.8087	0.8205	0.9848	0.8673	0.6977	1.0000
NCI-H1623	0.7226	0.7742	0.9082	0.8673	0.8148	0.8873
NCI-H1648	0.6001	0.7246	0.8400	0.8358	0.7000	0.9167
NCI-H1650	0.7084	0.7686	0.8836	0.8305	0.8333	0.8302

NCI-H1693	0.6717	0.7469	0.8801	0.7755	0.7500	0.7821
NCI-H1770	0.6737	0.7311	0.8599	0.7826	0.6333	0.8241
NCI-H1838	0.4514	0.6213	0.9328	0.7630	1.0000	0.7519
NCI-H2126	0.7951	0.7965	0.9453	0.8815	0.8235	0.9010
NCI-H2170	0.7797	0.8125	0.9451	0.8878	0.8125	0.9242
NCI-H2228	0.7591	0.8094	0.9525	0.8776	0.7500	0.9516
NCI-H2342	0.7128	0.7712	0.8453	0.7766	0.6250	0.8548
NCI-H2347	0.8389	0.8216	0.9476	0.8571	0.8750	0.8514
NCI-H2405	0.7488	0.7888	0.9259	0.8673	0.8065	0.8955
NCI-H661	0.7998	0.8226	0.9730	0.9490	0.9032	0.9701
DMS-114	0.6811	0.7492	0.8735	0.8261	0.7778	0.8378
DMS-273	0.7862	0.8111	0.9461	0.8571	0.6750	0.9828
NCI-H1048	0.7480	0.7911	0.9218	0.8571	0.7179	0.9492
NCI-H1092	0.6894	0.7412	0.8965	0.7826	0.7895	0.7815
NCI-H1417	0.8219	0.8179	0.9592	0.8354	0.9412	0.8065
NCI-H1694	0.7829	0.8030	0.9820	0.9275	0.9655	0.9174
NCI-H187	0.6990	0.7575	0.9222	0.8116	0.7333	0.8333
NCI-H1963	0.7934	0.8058	0.9672	0.9123	0.9063	0.9146
NCI-H2029	0.6448	0.7336	0.9891	0.7755	1.0000	0.7609
NCI-H209	0.6194	0.7268	0.8473	0.8160	0.6829	0.8810
NCI-H2141	0.7939	0.8022	0.9605	0.8986	0.9310	0.8899
NCI-H2171	0.6088	0.7196	0.7813	0.7468	0.7333	0.7500
NCI-H2227	0.6322	0.7130	0.8973	0.8230	0.8667	0.8163
NCI-H345	0.6772	0.7453	0.9393	0.7971	0.9333	0.7805
NCI-H446	0.7025	0.7530	0.9168	0.8551	0.8065	0.8692
NCI-H526	0.7711	0.7955	0.9483	0.8768	0.7442	0.9368
NCI-H64	0.7688	0.7936	0.9346	0.8861	0.9091	0.8772
NCI-H69	0.7397	0.7729	0.9275	0.8406	0.8333	0.8426
NCI-H82	0.6586	0.7347	0.8985	0.8478	0.7353	0.8846
NCI-H2052	0.7102	0.7734	0.9005	0.8265	0.8667	0.8088
SK-N-DZ	0.6048	0.7172	0.8041	0.7101	0.5532	0.7912
SK-N-FI	0.5618	0.6945	0.8947	0.8175	0.8421	0.8136
VA-ES-BJ	0.8348	0.8235	0.9476	0.8406	0.6833	0.9615
LU-139	0.6246	0.7379	0.8814	0.8480	0.7838	0.8750
SBC-5	0.7633	0.8071	0.9775	0.9184	0.8529	0.9531
SBC-1	0.6628	0.7321	0.8643	0.7810	0.7600	0.7857
LU-135	0.8142	0.8161	0.9773	0.8673	0.7045	1.0000
NCI-H2030	0.8264	0.8298	0.9408	0.8421	0.6829	0.9630
NCI-H2122	0.7490	0.7923	0.9094	0.8469	0.7381	0.9286
NCI-H1734	0.8350	0.8471	0.9598	0.9054	0.8333	0.9545
NCI-H650	0.8141	0.8134	0.9431	0.8673	0.8000	0.8971

T-24	0.6397	0.7294	0.8506	0.7755	0.6897	0.8116
SK-N-AS	0.6518	0.7313	0.8790	0.8265	0.7407	0.8592
NCI-H1299	0.7356	0.7911	0.9524	0.9027	0.7778	0.9610
NCI-H2087	0.7926	0.8050	0.9274	0.8571	0.7059	0.9375
UM-UC-3	0.7787	0.8204	0.9502	0.9043	0.7838	0.9825
SW756	0.7141	0.7739	0.8913	0.8646	0.7742	0.9077
NCI-H727	0.6902	0.7589	0.8649	0.8163	0.7308	0.8472
Calu-6	0.6865	0.7586	0.9082	0.8623	0.8286	0.8738
LU-65	0.5620	0.6675	0.9248	0.7826	0.7895	0.7815
NCI-H1355	0.7734	0.7978	0.9471	0.8696	0.7037	0.9762
NCI-H1792	0.7539	0.8003	0.9398	0.8776	0.8065	0.9104
HPAF-II	0.7288	0.7713	0.9427	0.8673	0.7742	0.9104
MIA-PaCa-2	0.7912	0.8214	0.9475	0.7857	0.6000	0.9792
SHP-77	0.6245	0.7334	0.8899	0.8188	0.8947	0.8067
NCI-H2009	0.8388	0.8464	0.9834	0.8763	0.7073	1.0000
NCI-H2291	0.3442	0.6169	0.5903	0.6633	0.2857	0.6923
SW1573	0.7518	0.8035	0.9832	0.9184	0.8929	0.9286
SW900	0.8036	0.8112	0.9611	0.9158	0.9286	0.9104
TE-5	0.7934	0.8195	0.9385	0.9203	0.8750	0.9388
HCC1954	0.7568	0.7747	0.9535	0.8061	0.9375	0.7805
HCC1143	0.7258	0.7685	0.9007	0.8454	0.8696	0.8378
HCC1187	0.6130	0.7361	0.8874	0.8116	0.7931	0.8165
HCC1395	0.6355	0.7385	0.9118	0.8265	0.9444	0.8000
HCC1599	0.7443	0.7920	0.8992	0.8861	0.7879	0.9565
HCC1937	0.7013	0.7915	0.9409	0.8469	0.8333	0.8514
HCC2157	0.7276	0.7694	0.8733	0.7971	0.6190	0.8750
HCC2218	0.5944	0.7081	0.8815	0.8188	0.7692	0.8304
HCC38	0.7296	0.7841	0.9426	0.8673	0.8889	0.8592
BB30-HNC	0.7739	0.8076	0.9175	0.7029	0.4744	1.0000
BB49-HNC	0.5489	0.7022	0.7876	0.7536	0.5588	0.8173
BB65-RCC	0.7888	0.8251	0.9395	0.8333	0.6500	0.9744
BEN	0.6861	0.7583	0.9533	0.8776	0.8750	0.8784
BHY	0.8207	0.8384	0.9474	0.8469	0.6591	1.0000
Ca9-22	0.8136	0.8191	0.9444	0.8878	0.7317	1.0000
CAL-12T	0.7580	0.8062	0.9516	0.8571	0.7073	0.9649
CAL-33	0.7662	0.7972	0.9317	0.8163	0.6875	0.9400
CP50-MEL-B	0.5777	0.7124	0.8276	0.7755	0.6786	0.8143
CP66-MEL	0.6484	0.7365	0.8896	0.8321	0.7429	0.8627
CPC-N	0.6998	0.7524	0.8794	0.8261	0.7222	0.8627
CTV-1	0.6501	0.7899	0.9191	0.7971	0.5970	0.9859
D-542MG	0.6023	0.7130	0.8400	0.7876	0.6923	0.8161

DMS-79	0.6092	0.7292	0.9061	0.7609	0.8889	0.7519
DSH1	0.6190	0.7235	0.8590	0.8043	0.6757	0.8515
EC-GI-10	0.5874	0.6937	0.8512	0.7899	0.7500	0.7982
EPLC-272H	0.8279	0.8264	0.9681	0.9082	0.8378	0.9508
HCE-4	0.7916	0.8161	0.9283	0.8699	0.7391	0.9481
HSC-2	0.8328	0.8355	0.9435	0.8571	0.6829	0.9825
IM-9	0.5493	0.6790	0.9208	0.7681	0.8824	0.7521
IST-SL1	0.7166	0.7646	0.8774	0.8406	0.7297	0.8812
IST-SL2	0.7372	0.8073	0.9224	0.8478	0.6667	0.9643
J82	0.7753	0.7930	0.9299	0.8511	0.7353	0.9167
KNS-62	0.7325	0.7726	0.9500	0.8596	0.6471	0.9500
KOSC-2	0.6631	0.7400	0.8743	0.7857	0.6774	0.8358
KP-4	0.7566	0.7939	0.9546	0.8830	0.8125	0.9194
KYSE-140	0.7797	0.8146	0.9310	0.8571	0.7000	0.9655
KYSE-410	0.8321	0.8380	0.9645	0.8367	0.6809	0.9804
KYSE-520	0.7712	0.7835	0.9162	0.8571	0.7333	0.9118
KYSE-70	0.7456	0.7934	0.9630	0.9286	0.9259	0.9296
LB1047-RCC	0.7307	0.7846	0.9160	0.7481	0.5323	0.9315
LB2241-RCC	NaN	0.6666	0.7553	0.6594	0.4068	0.8481
LB2518-MEL	0.6739	0.7647	0.8847	0.8478	0.7692	0.8788
LB373-MEL-D	0.6794	0.7533	0.9410	0.8686	0.8710	0.8679
LB647-SCLC	0.7530	0.7952	0.9062	0.7975	0.7143	0.8431
LB771-HNC	0.6428	0.7370	0.8215	0.7971	0.7097	0.8224
LB831-BLC	0.7268	0.7674	0.9066	0.8551	0.7000	0.9432
LB996-RCC	0.6256	0.7331	0.8564	0.7946	0.6522	0.8939
LCLC-103H	0.7363	0.7909	0.8883	0.8265	0.7097	0.8806
LU-134-A	0.7528	0.7759	0.9316	0.8478	0.7073	0.9072
LU-165	0.5723	0.6869	0.9517	0.7826	0.9167	0.7698
LXF-289	0.7641	0.8028	0.9432	0.7826	0.5522	1.0000
MS-1	0.7065	0.7582	0.9155	0.8406	0.7931	0.8532
MZ1-PC	0.7145	0.7709	0.8708	0.8188	0.6481	0.9286
MZ7-mel	0.6454	0.7159	0.8588	0.8116	0.6667	0.8687
NCI-H128	0.2567	0.5603	0.3333	0.7500	0.0000	0.7692
NCI-H1304	0.5933	0.7110	0.8725	0.8029	0.8000	0.8036
NCI-H1563	0.7057	0.7604	0.8784	0.7857	0.6800	0.8219
NCI-H292	0.7444	0.7782	0.9015	0.7857	0.5882	1.0000
NCI-H510A	0.7121	0.7652	0.9186	0.8696	0.8056	0.8922
PC-14	0.7928	0.8115	0.9256	0.8878	0.8056	0.9355
SK-MM-2	0.7127	0.7565	0.9108	0.8333	0.7333	0.8611
TE-15	0.7416	0.7796	0.8805	0.7681	0.5625	0.9459
U-266	0.6394	0.7306	0.8607	0.8600	0.6154	0.9459

SIMA	0.6557	0.7243	0.9096	0.8029	0.8966	0.7778
TE-1	0.7226	0.7610	0.9070	0.8768	0.7333	0.9462
TE-10	0.7732	0.7884	0.8961	0.8406	0.7059	0.9195
TE-8	0.8101	0.8074	0.9455	0.8333	0.6515	1.0000
CAPAN-1	0.7340	0.7858	0.9403	0.8878	0.8929	0.8857
OVCAR-3	0.7911	0.8191	0.9209	0.8367	0.8462	0.8333
PC-3	0.6736	0.7541	0.9509	0.8980	0.9032	0.8955
DU-145	0.5150	0.6714	0.7491	0.7041	0.5000	0.8448
HCT-116	0.7937	0.7997	0.9517	0.8878	0.7500	0.9828
HCT-15	0.7327	0.7757	0.8929	0.8163	0.6944	0.8871
HL-60	0.7500	0.7780	0.8854	0.7681	0.5556	0.9048
HT-29	0.7326	0.7711	0.9268	0.8061	0.6279	0.9455
K-562	0.4304	0.7126	0.8264	0.7971	0.6038	0.9176
NCI-H226	0.6978	0.7582	0.9183	0.8333	0.9130	0.8174
NCI-H23	0.6364	0.7262	0.8747	0.8333	0.7500	0.8585
NCI-H460	0.8440	0.8146	0.9350	0.7959	0.6042	0.9800
NCI-H522	0.7443	0.7899	0.9906	0.9444	0.8800	0.9787
T47D	0.6265	0.7259	0.8725	0.7959	0.7391	0.8133
MCF7	0.7244	0.7778	0.9187	0.8469	0.7073	0.9474
786-0	0.7371	0.7949	0.8945	0.7347	0.5294	0.9574
A498	0.2582	0.5931	0.6537	0.7045	0.5000	0.7455
A549	0.7738	0.7995	0.9333	0.7797	0.5000	0.9714
ACHN	0.7112	0.7909	0.8877	0.7766	0.6591	0.8800
BT-549	0.6845	0.7376	0.9224	0.8305	0.8000	0.8367
CCRF-CEM	0.7991	0.8118	0.9524	0.8043	0.6032	0.9733
SK-MEL-28	0.4868	0.6683	0.8188	0.7634	0.7143	0.7778
SK-MEL-2	0.6461	0.7235	0.9055	0.8478	0.7436	0.8889
SK-MEL-5	0.7079	0.7580	0.9097	0.8621	0.6111	0.9750
Hs-578-T	0.5912	0.7135	0.8301	0.7789	0.7037	0.8088
MOLT-4	0.8426	0.8327	0.9585	0.8731	0.7308	0.9634
SK-OV-3	0.8266	0.8214	0.9579	0.8980	0.8108	0.9508
MDA-MB-231	0.7572	0.7989	0.9467	0.8673	0.7778	0.9194
COLO-205	0.7051	0.7599	0.8698	0.7653	0.6000	0.8793
SW620	0.8367	0.8212	0.9664	0.8571	0.7250	0.9483
CAKI-1	0.7490	0.7871	0.9074	0.8061	0.7027	0.8689
RPMI-8226	0.6856	0.7548	0.9373	0.8613	0.6852	0.9759
SR	0.7542	0.8087	0.9439	0.8043	0.5873	0.9867
NCI-H322M	0.2890	0.5670	1.0000	0.7174	1.0000	0.7153
IGROV-1	0.7826	0.7767	0.9091	0.8475	0.6667	0.9091
OVCAR-5	0.7902	0.8060	0.9506	0.8842	0.8056	0.9322
EKVX	0.5606	0.7117	0.8273	0.7910	0.7500	0.8000

HCC2998	0.7078	0.7615	0.8967	0.8319	0.6744	0.9286
HOP-62	0.8040	0.8112	0.9277	0.8029	0.6032	0.9730
HOP-92	0.7814	0.7949	0.9167	0.8571	0.7105	0.9500
LOXIMVI	0.7545	0.7804	0.8895	0.8175	0.6415	0.9286
M14	0.6337	0.7402	0.9079	0.8776	0.7714	0.9365
UACC-62	0.6750	0.7334	0.9322	0.8776	0.8696	0.8800
UACC-257	0.6347	0.7324	0.8728	0.8406	0.6944	0.8922
RXF393	0.7209	0.7740	0.8597	0.7536	0.5472	0.8824
SN12C	0.7715	0.8027	0.9669	0.9286	0.8438	0.9697
TK10	0.8092	0.8187	0.9136	0.8261	0.6491	0.9506
U031	0.7167	0.7745	0.8919	0.8421	0.7143	0.9167
SNB75	0.7776	0.7976	0.9270	0.8986	0.8043	0.9457
U251	0.6853	0.7507	0.9015	0.8571	0.7813	0.8939
SF539	0.6444	0.7561	0.8762	0.8333	0.7143	0.8854
SF295	0.8023	0.8309	0.9450	0.8632	0.7500	0.9455
SF268	0.7888	0.8130	0.9354	0.8623	0.6909	0.9759
KM12	0.7245	0.7629	0.9153	0.8116	0.6271	0.9494
OVCAR-4	0.7746	0.7951	0.9005	0.8467	0.7021	0.9222
OVCAR-8	0.7884	0.8342	0.9674	0.9388	0.8788	0.9692
BxPC-3	0.7851	0.8043	0.9056	0.8265	0.6500	0.9483
BHT-101	0.6303	0.7330	0.8707	0.7959	0.6429	0.9107
Becker	0.7035	0.7698	0.9211	0.8333	0.6207	0.9875
BE-13	0.5839	0.7277	0.8476	0.8043	0.6094	0.9730
ARH-77	0.7162	0.7784	0.9450	0.9058	0.8611	0.9216
AGS	0.8161	0.8184	0.9479	0.7857	0.5957	0.9608
ABC-1	0.7402	0.7669	0.9043	0.8163	0.6571	0.9048
A2058	0.8582	0.8469	0.9858	0.9043	0.7568	1.0000
A375	0.7594	0.8071	0.9657	0.7959	0.6154	1.0000
A253	0.7532	0.8018	0.9538	0.9275	0.9000	0.9388
8305C	0.8174	0.8218	0.9375	0.8673	0.7500	0.9483
647-V	0.7567	0.8012	0.9957	0.9694	0.9375	0.9848
639-V	0.7666	0.7900	0.9141	0.8265	0.6591	0.9630
697	0.7806	0.7964	0.8568	0.6667	0.4691	0.9474
BT-20	0.7610	0.7801	0.9416	0.9082	0.8889	0.9155
ACN	0.7405	0.7917	0.9182	0.8478	0.6852	0.9524
A2780	0.7291	0.7942	0.9276	0.8673	0.7045	1.0000
COR-L105	0.7088	0.7652	0.8882	0.7857	0.6579	0.8667
CRO-AP2	0.7646	0.8024	0.9130	0.7612	0.4643	0.9744
COR-L88	0.6614	0.7472	0.9469	0.8406	0.9474	0.8235
COLO-824	0.6681	0.7287	0.9447	0.8188	0.9474	0.7983
COLO-800	0.5458	0.6867	0.8589	0.8116	0.6765	0.8558

COLO-792	0.6074	0.7017	0.7583	0.7143	0.5652	0.7600
COLO-741	0.7252	0.7618	0.8786	0.8163	0.7857	0.8286
COLO-680N	0.7791	0.8056	0.9438	0.8776	0.8636	0.8816
COLO-679	0.7763	0.7919	0.9243	0.8571	0.7073	0.9649
CHP-212	0.7497	0.7888	0.9257	0.7959	0.6275	0.9787
CFPAC-1	0.7918	0.8351	0.9574	0.8776	0.7813	0.9242
CCF-STTG1	0.7416	0.7884	0.8975	0.8247	0.6667	0.9180
Ca-Ski	0.8176	0.8285	0.9820	0.8776	0.7073	1.0000
Caov-3	0.7749	0.8327	0.9586	0.7692	1.0000	0.6897
CAL-120	0.7763	0.8033	0.9393	0.8776	0.8065	0.9104
CAL-72	0.5752	0.7200	0.8767	0.8163	0.7500	0.8378
CAL-62	0.7501	0.7940	0.9508	0.8571	0.7073	0.9649
C32	0.4870	0.6638	0.8126	0.7653	0.6400	0.8082
Daudi	0.7363	0.7716	0.8878	0.8406	0.6809	0.9231
DB	0.6730	0.7462	0.9490	0.8872	0.8095	0.9231
Daoy	0.7703	0.8113	0.9603	0.8980	0.7500	1.0000
D-283MED	0.5935	0.7077	0.8998	0.8478	0.8125	0.8585
DBTRG-05MG	0.8001	0.8069	0.9185	0.8061	0.6667	0.8871
DEL	0.6923	0.7595	0.9197	0.8333	0.6875	0.9111
Detroit562	0.8019	0.8048	0.9426	0.8878	0.7436	0.9831
DG-75	0.6320	0.7336	0.9017	0.8188	0.6829	0.8763
DK-MG	0.5133	0.6907	0.7878	0.7347	0.6000	0.7692
DJM-1	0.7447	0.7831	0.9761	0.9348	1.0000	0.9151
DOHH-2	0.6537	0.7525	0.8648	0.7674	0.6230	0.8971
DoTc2-4510	0.7723	0.8155	0.9552	0.8980	0.8387	0.9254
DU-4475	0.7759	0.8059	0.9344	0.8696	0.6842	1.0000
EB2	0.6045	0.6952	0.8821	0.8406	0.7742	0.8598
EB-3	0.6377	0.7265	0.9119	0.8261	0.8400	0.8230
ECC10	0.7585	0.7999	0.9213	0.8265	0.6429	0.9643
ECC12	0.5887	0.7249	0.8594	0.8214	0.5833	1.0000
EFM-19	0.6111	0.7509	0.8829	0.8265	0.6842	0.9167
EFO-27	0.8528	0.8346	0.9264	0.8571	0.7209	0.9636
EGI-1	0.7598	0.7892	0.9546	0.8673	0.7714	0.9206
EHEB	0.7731	0.8116	0.9504	0.8571	0.9048	0.8367
EM-2	0.3349	0.7104	0.7801	0.6884	0.4677	0.8684
EoL-1-cell	0.3636	0.7165	0.8449	0.7174	0.5000	0.9545
ETK-1	0.7805	0.8087	0.9431	0.8478	0.6441	1.0000
EVSA-T	0.6601	0.7472	0.8539	0.8321	0.7073	0.8854
FADU	0.7195	0.7774	0.9238	0.8673	0.7297	0.9508
FTC-133	0.6923	0.7418	0.9087	0.8367	0.7407	0.8732
G-361	0.7810	0.8075	0.9371	0.8673	0.7073	0.9825

GAMG	0.7926	0.8231	0.9481	0.8776	0.7143	1.0000
GCIY	0.7375	0.7766	0.9192	0.8333	0.7333	0.8817
GDM-1	0.4991	0.6777	0.8062	0.7391	0.5484	0.8947
GI-1	0.7261	0.7773	0.9199	0.8551	0.6981	0.9529
GI-ME-N	0.7106	0.7666	0.8997	0.8043	0.6364	0.9157
GMS-10	0.7041	0.7522	0.9386	0.8673	0.8000	0.8904
GOTO	0.7018	0.7701	0.8968	0.8623	0.8000	0.8835
GR-ST	0.6745	0.7568	0.8456	0.7826	0.5797	0.9855
GCT	NaN	0.7284	0.9125	0.8571	0.7576	0.9077
ESS-1	0.8075	0.8294	0.9301	0.8878	0.7941	0.9375
GT3TKB	0.7825	0.8109	0.9436	0.8841	0.7556	0.9462
H4	0.7492	0.7926	0.8956	0.7449	0.5556	0.9773
H9	0.7403	0.7762	0.9170	0.8551	0.6939	0.9438
HC-1	0.7660	0.8013	0.8943	0.8623	0.7692	0.8990
HCC1419	0.5410	0.6726	0.8696	0.7653	0.7273	0.7763
HCC1569	0.6931	0.7562	0.9419	0.8571	0.8400	0.8630
HCC1806	0.7459	0.8039	0.9710	0.9286	0.8529	0.9688
HCC70	0.7382	0.7715	0.8804	0.8061	0.6333	0.8824
HD-MY-Z	0.7310	0.7812	0.9257	0.8433	0.6667	0.9625
HEC-1	0.8309	0.8351	0.9946	0.9388	0.9333	0.9412
HEL	0.7812	0.7950	0.9385	0.8188	0.6333	0.9615
HGC-27	0.7692	0.7984	0.9500	0.8265	0.6458	1.0000
HH	0.7758	0.7935	0.9003	0.8333	0.6538	0.9419
HLE	0.8048	0.8178	0.9315	0.8469	0.7250	0.9310
HMV-II	0.7897	0.8100	0.9286	0.8652	0.7273	0.9464
HN	0.6850	0.7465	0.8781	0.7959	0.6316	0.9000
HOS	0.7953	0.8329	0.9753	0.8265	0.6444	0.9811
HSC-3	0.8063	0.8138	0.9500	0.8061	0.6122	1.0000
HSC-4	0.6397	0.7442	0.8802	0.8243	0.7500	0.8810
HT	0.7718	0.7882	0.9422	0.8913	0.7907	0.9368
HT-1080	0.7468	0.7949	0.9217	0.7755	0.5918	0.9592
HT-1197	0.6982	0.7681	0.9446	0.8265	0.9333	0.8072
HT-1376	0.7929	0.8064	0.9396	0.8571	0.8846	0.8472
HT-144	0.7793	0.7989	0.9143	0.8116	0.6032	0.9867
HT-3	NaN	0.7859	0.9501	0.8763	0.8214	0.8986
HuCCT1	0.8371	0.8256	0.9365	0.8469	0.6923	0.9492
HuH-7	0.7477	0.7959	0.8741	0.8061	0.6389	0.9032
HuO9	0.4837	0.6724	0.8175	0.7551	0.6129	0.8209
HUTU-80	0.7304	0.8023	0.9411	0.8116	0.6515	0.9583
IGR-1	0.7908	0.8189	0.9207	0.8673	0.7576	0.9231
IMR-5	0.6563	0.7240	0.9009	0.8478	0.7500	0.8824

IPC-298	0.7183	0.7694	0.9331	0.8776	0.7568	0.9508
IST-MEL1	0.7902	0.8239	0.9419	0.8696	0.7255	0.9540
IST-MES1	0.5959	0.7130	0.8381	0.7971	0.7188	0.8208
JAR	0.5824	0.6963	0.9168	0.8613	0.8519	0.8636
JEG-3	0.7594	0.7917	0.9892	0.9592	0.9333	0.9706
JiyoyeP-2003	0.7601	0.7917	0.9553	0.9130	0.7917	0.9778
JVM-2	0.5758	0.6943	0.8330	0.7609	0.6667	0.7807
JVM-3	0.4806	0.6605	0.7883	0.7464	0.5667	0.7963
KALS-1	0.7967	0.8008	0.9148	0.8582	0.7143	0.9412
KARPAS-45	0.7202	0.7768	0.8903	0.8102	0.6531	0.8977
KARPAS-299	0.7115	0.7663	0.8923	0.8406	0.7436	0.8788
KARPAS-422	0.7756	0.7882	0.8975	0.8261	0.6275	0.9425
KASUMI-1	0.4118	0.6476	0.7773	0.7386	0.5417	0.8125
KE-37	0.7722	0.7949	0.9415	0.8043	0.5833	0.9744
KG-1	0.5444	0.7053	0.8252	0.7971	0.6047	0.8842
KINGS-1	0.6600	0.7404	0.8679	0.8116	0.6944	0.8529
KMOE-2	0.7161	0.7711	0.8690	0.8043	0.6429	0.8750
KNS-42	0.7035	0.7602	0.9341	0.8768	0.9286	0.8636
HuP-T3	0.7949	0.8035	0.9194	0.8571	0.8214	0.8714
HuP-T4	0.8089	0.8085	0.9754	0.8878	0.7500	0.9828
HT55	0.7072	0.7618	0.8418	0.7174	0.7273	0.7160
H-EMC-SS	0.5029	0.6809	0.7593	0.6934	0.5250	0.7629
GP5d	0.7854	0.7883	0.9494	0.8878	0.8387	0.9104
DMS-53	0.5544	0.6950	0.9441	0.8041	0.9231	0.7857
G-402	0.6980	0.7768	0.9083	0.8265	0.6667	0.9800
G-401	0.8057	0.8165	0.9337	0.7857	0.6170	0.9412
KY821	0.5866	0.7079	0.8100	0.7737	0.6897	0.7963
KU812	0.7185	0.7696	0.8747	0.7754	0.5593	0.9367
KU-19-19	0.6269	0.7235	0.8894	0.7857	0.7727	0.7895
KS-1	0.7288	0.7686	0.8926	0.7536	0.5571	0.9559
KP-N-YN	0.6056	0.7203	0.8469	0.7754	0.7826	0.7739
KYSE-150	0.7553	0.7980	0.9209	0.8061	0.6410	0.9153
KYSE-180	0.7393	0.7852	0.8746	0.8367	0.6829	0.9474
KYSE-270	0.8242	0.8174	0.9514	0.8673	0.7209	0.9818
KYSE-450	0.7860	0.8157	0.9430	0.8866	0.7250	1.0000
KYSE-510	0.7658	0.8277	0.9444	0.8469	0.6829	0.9649
L-428	0.7282	0.7664	0.9361	0.8768	0.8667	0.8796
L-540	0.7892	0.8114	0.9462	0.8913	0.7778	0.9462
LAMA-84	0.4689	0.7110	0.8523	0.7101	0.5139	0.9242
LC-2-ad	0.7674	0.7946	0.9102	0.8478	0.6613	1.0000
LC4-1	0.7099	0.7585	0.9020	0.8406	0.7500	0.8725

LNCaP-Clone-FGC	0.6677	0.7184	0.9024	0.8406	0.7647	0.8654
LOUCY	0.7344	0.7924	0.9229	0.8478	0.7059	0.9310
LoVo	0.7653	0.7900	0.9074	0.7755	0.5957	0.9412
LP-1	0.5942	0.7033	0.8749	0.7971	0.7826	0.8000
LS-123	0.7597	0.7849	0.9334	0.8768	0.7949	0.9091
LS-411N	0.7770	0.7895	0.9517	0.8623	0.6964	0.9756
LS-513	0.6674	0.7367	0.8863	0.8188	0.7742	0.8318
LU-99A	0.7762	0.8193	0.9555	0.8673	0.8621	0.8696
MC116	0.6324	0.7123	0.8713	0.8261	0.7143	0.8750
MC-IXC	0.7145	0.7726	0.8814	0.8367	0.6667	0.9643
MDA-MB-175-VII	0.6745	0.7487	0.8563	0.7895	0.7391	0.8056
MDA-MB-361	0.7944	0.7940	0.9510	0.8776	0.7714	0.9365
MDA-MB-453	0.6338	0.7212	0.8689	0.7959	0.8750	0.7805
MDA-MB-468	0.6828	0.7485	0.8847	0.8041	0.6774	0.8636
MEL-HO	0.7536	0.7808	0.9010	0.8136	0.6190	0.9211
MEL-JUSO	0.7704	0.8041	0.9474	0.8265	0.6383	1.0000
MEG-01	0.5260	0.7284	0.8413	0.7754	0.6000	0.9315
MES-SA	0.7554	0.8011	0.9251	0.8454	0.6667	0.9818
Mewo	0.6507	0.7280	0.8819	0.8469	0.7667	0.8824
MFE-280	0.7041	0.7700	0.8988	0.7857	0.8000	0.7831
MFE-296	0.7926	0.8214	0.9044	0.8367	0.6744	0.9636
MG-63	0.7212	0.7766	0.9043	0.8163	0.6500	0.9310
MHH-CALL-2	0.6511	0.7306	0.9131	0.8644	0.7500	0.8936
MHH-ES-1	0.7294	0.8018	0.9192	0.8367	0.6939	0.9796
MHH-NB-11	0.6839	0.7537	0.8862	0.8045	0.7059	0.8384
MHH-PREB-1	0.7661	0.7898	0.9240	0.8696	0.7255	0.9540
MKN1	0.7539	0.8039	0.9554	0.8830	0.8621	0.8923
MKN28	0.7551	0.8176	0.9610	0.8980	0.8056	0.9516
ML-2	0.7349	0.7665	0.9042	0.7899	0.5893	0.9268
MLMA	0.7620	0.7932	0.9237	0.8478	0.6731	0.9535
MN-60	0.7536	0.7782	0.9647	0.8478	0.9545	0.8276
MOLT-13	0.6653	0.7333	0.9213	0.8267	0.5926	0.9583
MOLT-16	0.7097	0.7638	0.8860	0.6852	0.4231	0.9286
MONO-MAC-6	0.7347	0.7973	0.9216	0.7754	0.5441	1.0000
Mo-T	0.5231	0.6744	0.8402	0.7681	0.5882	0.8269
MPP-89	0.6809	0.7576	0.9007	0.8406	0.6875	0.9222
MRK-nu-1	0.5913	0.7196	0.8110	0.8116	0.6744	0.8737
MSTO-211H	0.4727	0.6448	0.7933	0.7445	0.6087	0.7719
MV-4-11	0.5718	0.7552	0.8547	0.5985	0.4138	0.9200
NALM-6	0.7722	0.7965	0.9181	0.7810	0.5821	0.9714
NB69	0.6841	0.7461	0.8670	0.7536	0.6207	0.8500

NCI-H520	0.7726	0.8016	0.9302	0.8980	0.7714	0.9683
NCI-SNU-1	0.6046	0.7253	0.8877	0.8043	0.8500	0.7966
NCI-SNU-5	0.6881	0.7548	0.8653	0.8333	0.7436	0.8687
NCI-SNU-16	0.3926	0.6263	0.8010	0.7826	0.6786	0.8091
NH-12	0.6641	0.7315	0.8852	0.8188	0.7500	0.8469
NKM-1	0.7105	0.7863	0.8904	0.7391	0.5156	0.9324
NMC-G1	0.7750	0.7979	0.9284	0.8841	0.7400	0.9659
no-11	0.7462	0.7813	0.9434	0.8768	0.8788	0.8762
NOMO-1	0.7105	0.7765	0.9234	0.7826	0.5660	0.9176
no-10	0.6021	0.7112	0.8294	0.8261	0.7429	0.8544
NUGC-3	0.7888	0.8024	0.9138	0.8163	0.6750	0.9138
NCI-H747	0.7932	0.7876	0.8704	0.8029	0.6327	0.8977
NCI-H716	0.2364	0.5904	0.6193	0.6842	0.5000	0.7386
NCI-H596	0.7876	0.8010	0.9715	0.8776	1.0000	0.8500
NCI-H441	0.8091	0.8166	0.9271	0.8454	0.8261	0.8514
NCI-N87	0.6983	0.7580	0.9242	0.8644	0.7273	0.8958
NCI-H2452	0.4897	0.6642	0.8552	0.7041	0.8125	0.6829
NCI-H1793	0.6515	0.7393	0.8987	0.8571	0.8947	0.8481
NCI-H358	0.7721	0.7950	0.9106	0.8511	0.7273	0.9180
NCI-H1155	0.5893	0.7161	0.9088	0.8248	0.7576	0.8462
NCI-H1436	0.5469	0.6930	0.7832	0.7949	0.7895	0.7966
NCI-H28	0.7507	0.7865	0.9075	0.8265	0.7241	0.8696
NCI-H1581	0.6019	0.7320	0.9008	0.8768	0.8387	0.8879
NCI-H1573	0.6045	0.7111	0.8656	0.8061	0.9286	0.7857
NCI-H1666	0.4846	0.6421	0.8234	0.7537	0.6667	0.7699
NCI-H1703	0.6182	0.7593	0.8670	0.8469	0.7179	0.9322
NCI-H1755	0.6970	0.7637	0.8846	0.8265	0.7241	0.8696
NCI-H1993	0.3901	0.5965	0.8377	0.7755	0.7778	0.7753
NCI-H2081	0.8205	0.8208	0.9464	0.8101	1.0000	0.7727
NCI-H2196	0.7396	0.8010	0.9868	0.8354	1.0000	0.8000
NCI-H630	0.7230	0.7390	0.9153	0.8472	0.8889	0.8413
NCI-H524	0.6692	0.7394	0.9053	0.8406	0.7419	0.8692
NCI-H748	0.7528	0.7822	0.9752	0.8608	1.0000	0.8254
ONS-76	0.7741	0.7910	0.9221	0.8116	0.6102	0.9620
OPM-2	0.6348	0.7274	0.8155	0.7826	0.6316	0.8400
OS-RC-2	0.7240	0.7776	0.8694	0.7681	0.5833	0.9103
P12-ICHIKAWA	0.7276	0.7679	0.9375	0.8776	0.7879	0.9231
P30-OHK	0.6531	0.7326	0.8603	0.7826	0.6136	0.8617
P31-FUJ	0.5165	0.6454	0.9335	0.7754	0.9231	0.7600
PA-1	0.8098	0.8275	0.9341	0.8469	0.7143	0.9464
OCUB-M	0.6828	0.7607	0.8611	0.7971	0.6000	0.9277

OC-314	0.8220	0.8252	0.9727	0.9322	0.8000	0.9773
PF-382	0.7980	0.7948	0.9114	0.8478	0.6786	0.9634
Raji	0.7479	0.7805	0.9121	0.8768	0.8438	0.8868
RCM-1	0.7455	0.7852	0.9234	0.8061	0.8182	0.8026
RD	0.7882	0.7995	0.9287	0.8776	0.8182	0.9077
REH	0.7307	0.7657	0.9084	0.8406	0.7368	0.8800
RKO	0.8402	0.8421	0.9540	0.7754	0.5797	0.9710
RMG-I	0.5859	0.7478	0.9020	0.7368	0.8235	0.7179
RPMI-2650	0.7895	0.8151	0.9536	0.8776	0.7941	0.9219
RPMI-6666	0.7653	0.8139	0.8920	0.8333	0.7000	0.9091
RPMI-8402	0.7965	0.8044	0.9102	0.8467	0.6604	0.9643
RS4-11	0.7174	0.7803	0.8834	0.7246	0.5072	0.9420
RT-112	0.7993	0.8174	0.9224	0.7755	0.6078	0.9574
RVH-421	0.7126	0.7742	0.9229	0.8367	0.7429	0.8889
Saos-2	0.7391	0.7963	0.9520	0.8673	0.7857	0.9000
SAS	0.7050	0.7505	0.9153	0.8367	0.7419	0.8806
SCC-9	0.6563	0.7429	0.9116	0.8265	0.9048	0.8052
SCH	0.4144	0.6287	0.9016	0.7652	1.0000	0.7438
SF126	0.7653	0.7860	0.9185	0.8261	0.6271	0.9747
SH-4	0.7067	0.7621	0.8575	0.7899	0.5932	0.9367
SIG-M5	0.6938	0.7955	0.8956	0.7215	0.5532	0.9688
SJRH30	0.7905	0.8073	0.9775	0.9381	0.9355	0.9394
SJSA-1	0.8389	0.8283	0.9425	0.8551	0.6780	0.9873
SK-CO-1	0.7685	0.7966	0.9581	0.8632	0.7647	0.9180
SK-HEP-1	0.8027	0.8205	0.9310	0.8265	0.6750	0.9310
SK-LMS-1	0.7457	0.7959	0.9013	0.7591	0.5652	0.9559
SK-LU-1	0.8081	0.8266	0.9343	0.8163	0.6364	0.9630
SKM-1	0.3516	0.6289	0.6983	0.6364	0.3243	0.8226
SK-MEL-1	0.4750	0.6660	0.7606	0.7101	0.5833	0.7222
SK-MEL-3	0.7390	0.7730	0.9054	0.8571	0.7333	0.9118
SK-MEL-24	0.5103	0.6636	0.8400	0.7527	0.7368	0.7568
SK-MEL-30	0.7327	0.7850	0.9063	0.8454	0.6757	0.9500
SK-MES-1	0.7888	0.8279	0.9375	0.8265	0.6667	0.9623
SK-MG-1	0.7975	0.8209	0.9254	0.8621	0.6190	1.0000
SK-NEP-1	0.7789	0.7958	0.9454	0.8478	0.6792	0.9529
SK-PN-DW	0.7722	0.8108	0.9821	0.8647	0.6786	1.0000
SK-UT-1	0.7790	0.8126	0.9330	0.8372	0.6667	0.9722
SNG-M	0.7568	0.8050	0.9118	0.8571	0.7297	0.9344
SNU-387	0.7965	0.7983	0.9390	0.8557	0.7500	0.9298
SNU-423	0.7838	0.8110	0.9390	0.8478	0.7179	0.9434
SNU-449	0.7495	0.7859	0.9113	0.8351	0.7931	0.8529

SNU-475	0.7029	0.7787	0.9714	0.6410	1.0000	0.6000
SNU-C2B	0.8195	0.8162	0.9224	0.8862	0.8108	0.9186
SU-DHL-1	0.6298	0.6897	0.8803	0.8354	0.7917	0.8545
SUP-T1	0.4943	0.6377	0.8592	0.7971	0.6667	0.8381
SW13	0.7617	0.7955	0.9334	0.8936	0.8182	0.9344
SW1088	0.6387	0.7229	0.8470	0.7449	0.6176	0.8125
SW1116	0.3325	0.6090	0.7738	0.7797	0.3333	0.8036
SW1417	0.6666	0.7467	0.9149	0.8163	0.8667	0.8072
SW1463	0.6501	0.7433	0.9158	0.7653	0.8462	0.7529
SW1710	0.7958	0.8203	0.9491	0.7653	0.5490	1.0000
SW1783	0.7600	0.7995	0.9627	0.8980	0.8667	0.9118
SW48	0.8339	0.8254	0.9655	0.8776	0.7500	0.9655
SW626	0.8214	0.8405	0.9797	0.9158	0.8824	0.9344
SW684	0.4473	0.6634	0.7604	0.7464	0.6667	0.7561
SW837	0.7654	0.8117	0.9583	0.8723	0.9048	0.8630
SW872	0.8015	0.8047	0.9331	0.7826	0.5902	0.9351
SW948	0.6151	0.7164	0.9486	0.7857	1.0000	0.7558
SW962	0.7516	0.7802	0.8975	0.8478	0.6939	0.9326
SW982	0.7845	0.7990	0.9090	0.7561	0.5538	0.9828
T84	0.6074	0.7036	0.8696	0.7857	0.6957	0.8133
TALL-1	0.5945	0.7183	0.8912	0.7821	1.0000	0.7500
TGBC1TKB	0.7340	0.7810	0.9175	0.8623	0.7778	0.8922
TGBC11TKB	0.7468	0.7660	0.9071	0.8469	0.8750	0.8378
THP-1	0.6110	0.7151	0.9390	0.8333	0.9167	0.8158
TUR	0.6688	0.7306	0.9169	0.8406	0.7143	0.8958
TYK-nu	0.6646	0.7485	0.8675	0.7789	0.6316	0.8772
U-2-OS	0.4887	0.6672	0.7835	0.6939	0.5185	0.7606
U-698-M	0.6780	0.7499	0.9466	0.8841	0.8611	0.8922
UACC-893	0.6148	0.7136	0.8197	0.7553	0.7647	0.7532
UMC-11	0.6810	0.7490	0.9041	0.7755	0.7647	0.7778
VM-CUB-1	0.7991	0.8228	0.9599	0.8776	0.7250	0.9828
VMRC-RCZ	0.7860	0.8216	0.9332	0.8878	0.7778	0.9516
WM-115	0.4158	0.6463	0.7699	0.7143	0.5200	0.7808
WSU-NHL	0.6599	0.7357	0.8824	0.8261	0.6905	0.8854
YAPC	0.6517	0.7248	0.8591	0.7653	0.8000	0.7614
YH-13	0.7059	0.7557	0.9385	0.8305	0.5500	0.9744
ZR-75-30	0.6336	0.7253	0.8302	0.7759	0.6000	0.7925
RCC10RGB	0.5319	0.6743	0.8386	0.7609	0.7500	0.7623
KURAMOCHI	0.7873	0.8017	0.9109	0.8406	0.6875	0.9222
KM-H2	0.6898	0.7487	0.9175	0.8841	0.9286	0.8727
HuO-3N1	0.6257	0.7288	0.9209	0.8367	0.8462	0.8333

OE19	0.7458	0.7827	0.9052	0.8673	0.8800	0.8630
NCI-H838	0.7655	0.7906	0.9320	0.8421	0.7353	0.9016
Ramos-2G6-4C10	0.7050	0.7773	0.9334	0.8623	0.6981	0.9647
RPMI-8866	0.5587	0.6925	0.8120	0.7609	0.5490	0.8851
QIMR-WIL	0.7800	0.8182	0.9096	0.8261	0.6250	0.9634
PSN1	0.7353	0.7780	0.8801	0.7754	0.5763	0.9241
OAW-42	0.8128	0.8213	0.9553	0.8830	0.8788	0.8852
OE33	0.8357	0.8292	0.9596	0.8776	0.8387	0.8955
CW-2	0.3163	0.5599	0.8194	0.7319	0.8333	0.7273
CMK	0.7697	0.7975	0.9112	0.8116	0.6034	0.9625
CHP-126	0.7270	0.7674	0.9034	0.7971	0.7297	0.8218
CGTH-W-1	0.5800	0.7211	0.8596	0.7899	0.6000	0.9359
COLO-320-HSR	0.7411	0.7618	0.9572	0.8768	0.7273	0.9468
ATN-1	0.6868	0.7707	0.8775	0.8116	0.6364	0.9277
CESS	0.7550	0.8012	0.8988	0.8551	0.7143	0.9167
COLO-678	0.6584	0.7330	0.8889	0.7653	0.7647	0.7654
COLO-684	0.8162	0.7997	0.9388	0.8768	0.7609	0.9348
COLO-668	0.6735	0.7725	0.9228	0.8333	0.8400	0.8319
LN-405	0.6145	0.7185	0.8941	0.8367	0.7727	0.8553
TGBC24TKB	0.6508	0.7344	0.8529	0.7886	0.6757	0.8372
A388	0.7563	0.7877	0.8916	0.8623	0.6727	0.9880
BFTC-909	0.7733	0.8157	0.9779	0.9184	0.8235	0.9688
C2BBe1	0.6891	0.7548	0.8526	0.8116	0.6667	0.8817
SCC-25	0.8010	0.8237	0.9449	0.8673	0.7500	0.9355
AsPC-1	0.6804	0.7467	0.9060	0.8061	0.7308	0.8333
CA46	0.7019	0.7562	0.8969	0.8478	0.7931	0.8624
AU565	0.6839	0.7658	0.9052	0.8163	0.6444	0.9623
BALL-1	0.7317	0.7680	0.9530	0.8983	0.7333	0.9545
BL-41	0.8143	0.8093	0.9385	0.8406	0.6613	0.9868
BL-70	0.6257	0.8007	0.9383	0.7595	0.6136	0.9429
BV-173	0.4773	0.7078	0.8005	0.6884	0.4861	0.9091
IA-LM	0.7515	0.7965	0.8979	0.8571	0.7083	0.9577
TGW	0.8064	0.8181	0.9125	0.8321	0.7632	0.8586
AN3-CA	0.7400	0.8030	0.9173	0.7835	0.6078	0.9783
A204	0.7098	0.7780	0.9043	0.8469	0.7174	0.9615
NY	0.7468	0.7984	0.9273	0.8367	0.6829	0.9474
C3A	0.7115	0.7681	0.9284	0.8469	0.8462	0.8472
A427	0.7694	0.8207	0.9101	0.8163	0.6304	0.9808
CAL-85-1	0.7160	0.7721	0.8670	0.8144	0.6667	0.9016
CHL-1	0.8029	0.8197	0.9209	0.7959	0.5918	1.0000
RL	0.4539	0.6129	0.8407	0.7754	0.7500	0.7787

NCI-H1651	0.7450	0.7764	0.9005	0.8571	0.7931	0.8841
RPMI-7951	0.7956	0.8241	0.9713	0.8673	0.6923	0.9831
SCC-4	0.7764	0.8058	0.9701	0.9184	0.8000	0.9841
SNU-C1	0.4733	0.6466	0.7921	0.7391	0.5556	0.7667
ST486	0.7322	0.7794	0.8635	0.8188	0.6296	0.9405
SW1990	0.7760	0.8214	0.9510	0.8878	0.9286	0.8714
UACC-812	0.6124	0.7034	0.9554	0.7167	1.0000	0.6964
SCC-15	0.6377	0.7370	0.9166	0.8582	0.8182	0.8713
Capan-2	0.6929	0.7742	0.9375	0.8367	0.8889	0.8250
CAL-27	0.7294	0.7780	0.9157	0.8571	0.7073	0.9649
BC-3	0.8109	0.8290	0.9364	0.8481	0.7143	0.9545
BC-1	0.7143	0.7721	0.9071	0.8478	0.7174	0.9130
A704	0.5757	0.7142	0.8262	0.7544	0.6111	0.8205
A101D	0.7972	0.8270	0.9470	0.8116	0.6338	1.0000
769-P	0.7448	0.8045	0.8827	0.8367	0.6923	0.9322
23132-87	0.8122	0.8114	0.9473	0.8763	0.7838	0.9333
A431	0.8259	0.8479	0.9748	0.9082	0.7805	1.0000
BFTC-905	0.7827	0.8304	0.9588	0.7857	0.5800	1.0000
CAL-51	0.7548	0.8176	0.9335	0.8367	0.6818	0.9630
SCC-3	0.6008	0.7303	0.8405	0.8101	0.7407	0.8462
RERF-LC-MS	0.6661	0.7484	0.9527	0.8776	0.7813	0.9242
GAK	0.6879	0.7533	0.9029	0.8258	0.7742	0.8416
AM-38	0.6589	0.7464	0.9405	0.8623	0.8966	0.8532
A4-Fuk	0.7589	0.7872	0.8868	0.7391	0.4923	0.9589
A3-KAW	0.7258	0.7642	0.8924	0.8406	0.6727	0.9518
DOK	0.5333	0.6825	0.7744	0.6735	0.5128	0.7797
COR-L279	0.7043	0.7604	0.8696	0.8321	0.7500	0.8614
CHP-134	0.4557	0.6486	0.8908	0.7917	0.2857	1.0000
NEC8	0.7373	0.7798	0.8824	0.8309	0.6863	0.9176
CAS-1	0.6398	0.7321	0.8131	0.7536	0.6765	0.7788
ALL-PO	0.6721	0.7586	0.8494	0.6812	0.4487	0.9833
S-117	0.6120	0.7324	0.8236	0.7449	0.6552	0.7826
OCI-AML2	0.7149	0.7806	0.9070	0.7971	0.5893	0.9390
MFM-223	0.4180	0.6146	0.8131	0.7899	0.7500	0.7966
CAL-54	0.6919	0.7461	0.8621	0.7959	0.6471	0.8750
EFO-21	0.7920	0.7902	0.8964	0.8571	0.8000	0.8767
LS-1034	0.7617	0.7830	0.9130	0.8478	0.7667	0.8704
22RV1	0.8261	0.8281	0.9858	0.9388	0.9333	0.9412
8505C	0.8079	0.8353	0.9691	0.8557	0.7073	0.9643
BCPAP	0.7744	0.8134	0.9312	0.8163	0.6596	0.9608
BPH-1	0.7094	0.7616	0.8515	0.7755	0.5957	0.9412

CAL-148	0.7887	0.7905	0.9502	0.9231	0.9565	0.9091
CAL-39	0.7814	0.8109	0.9255	0.8061	0.6136	0.9630
CaR-1	0.6987	0.7633	0.9474	0.8673	0.9048	0.8571
HDLM-2	0.6788	0.7545	0.9053	0.8478	0.8235	0.8558
HO-1-N-1	0.7555	0.7772	0.9091	0.8367	0.6750	0.9483
HTC-C3	0.7320	0.7696	0.9174	0.8061	0.6098	0.9474
KGN	0.7586	0.8086	0.9026	0.7754	0.6032	0.9200
KLE	0.7472	0.7942	0.9033	0.8130	0.8462	0.8041
KNS-81-FD	0.6749	0.7404	0.9176	0.8261	0.8148	0.8288
KP-N-RT-BM-1	0.5549	0.7000	0.7937	0.8000	0.7143	0.8333
K5	0.7892	0.8039	0.9171	0.8295	0.6818	0.9059
L-363	0.7721	0.7961	0.9237	0.8333	0.6538	0.9419
MDA-MB-415	0.6776	0.7348	0.8750	0.8242	0.7778	0.8438
NCI-H1975	0.7976	0.8108	0.9327	0.8015	0.6061	0.9857
SW954	0.7768	0.7952	0.9047	0.8116	0.6129	0.9737
TE-441-T	0.6425	0.7491	0.9384	0.8481	0.8571	0.8448
MKN7	0.6760	0.7522	0.9911	0.9492	1.0000	0.9400
MDA-MB-157	0.6256	0.7151	0.9061	0.7959	0.8421	0.7848
MMAC-SF	0.6937	0.7598	0.9297	0.8605	0.8235	0.8737
MKN45	0.7563	0.7972	0.9529	0.9101	0.8125	0.9649
NCI-H810	0.7170	0.7675	0.9029	0.8469	0.6857	0.9365
NBsusSR	0.7635	0.7955	0.9368	0.8261	0.6230	0.9870
MFH-ino	0.7775	0.8012	0.9241	0.8116	0.6000	1.0000
NOS-1	0.6560	0.7524	0.8556	0.7754	0.5606	0.9722
PANC-03-27	0.8348	0.8388	0.9439	0.8469	0.7073	0.9474
PANC-08-13	0.6652	0.7427	0.9285	0.8673	0.8333	0.8784
PANC-10-05	0.7994	0.8109	0.9399	0.8469	0.7813	0.8788
RL95-2	0.7467	0.7769	0.9018	0.7864	0.5455	0.9661
RO82-W-1	0.6759	0.7461	0.8690	0.7347	0.7857	0.7262
SiHa	0.5851	0.6887	0.8893	0.7895	0.9231	0.7683
SKG-IIIa	0.7541	0.7831	0.9292	0.8660	0.8750	0.8630
TT	0.5066	0.6741	0.8064	0.7857	0.7000	0.8077
TE-9	0.7476	0.7868	0.9202	0.8841	0.7778	0.9355
TE-11	0.7586	0.7824	0.8922	0.8130	0.6522	0.9091
TE-6	0.7240	0.7806	0.9152	0.8696	0.8235	0.8846
TE-12	0.6952	0.7452	0.8396	0.7391	0.5323	0.9079
TC-YIK	0.6856	0.7459	0.9259	0.8636	0.6111	0.9583
YT	0.4848	0.6489	0.8142	0.7319	0.5349	0.8211
BT-474	0.6188	0.7291	0.9045	0.8333	0.8077	0.8393
OAW-28	0.6938	0.7431	0.9362	0.8367	0.8696	0.8267
LCLC-97TM1	0.7947	0.8193	0.9312	0.8265	0.6444	0.9811

KP-N-YS	0.7005	0.7553	0.9062	0.8450	0.7353	0.8842
D-247MG	0.5840	0.7109	0.7894	0.8125	0.6000	0.8736
D-263MG	0.6448	0.7314	0.8275	0.7246	0.5122	0.8144
D-336MG	0.5408	0.6859	0.7454	0.7282	0.4571	0.8676
D-392MG	0.5822	0.7070	0.8738	0.7876	0.7778	0.7907
D-423MG	0.7352	0.7814	0.9309	0.8644	0.7222	0.9268
D-502MG	0.4523	0.6476	0.8021	0.7464	0.6364	0.7672
D-566MG	0.7594	0.7989	0.9202	0.7627	0.4583	0.9714
CAMA-1	0.7426	0.7753	0.9384	0.8776	0.8387	0.8955
CTB-1	0.7405	0.8039	0.9215	0.8696	0.7037	0.9762
Caov-4	0.6519	0.7350	0.8383	0.7835	0.7222	0.7975
CP67-MEL	0.7989	0.7933	0.9097	0.8400	0.6667	0.9375
M059J	0.7369	0.7793	0.9133	0.8673	0.7273	0.9385
HAL-01	0.6985	0.7632	0.8890	0.7391	0.5323	0.9079
OMC-1	0.7668	0.7779	0.9364	0.8712	0.8571	0.8763
ES8	0.7937	0.8085	0.9523	0.8478	0.6786	0.9634
ES4	0.7634	0.8196	0.9304	0.8116	0.6290	0.9605
ES6	0.6984	0.7652	0.8895	0.8116	0.6154	0.9302
ES1	0.6937	0.7637	0.9139	0.8696	0.7647	0.9310
EW-7	0.7382	0.8037	0.9181	0.8367	0.7188	0.8939
EW-3	0.7761	0.7854	0.8816	0.8333	0.7143	0.8738
EW-1	0.7464	0.7831	0.9585	0.8913	0.7170	1.0000
EW-18	0.5983	0.7113	0.9140	0.8551	0.7714	0.8835
EW-16	0.7757	0.8166	0.9513	0.8116	0.6471	0.9714
EW-13	0.5687	0.7244	0.8401	0.8116	0.7576	0.8286
EW-22	0.6546	0.7205	0.8782	0.8378	0.5625	0.9138
EW-24	0.5815	0.7216	0.7880	0.7609	0.7059	0.7686
LAN-6	0.6717	0.7355	0.8566	0.7899	0.7059	0.8173
NB10	0.6359	0.7261	0.8903	0.8116	0.6842	0.8600
NB12	0.6994	0.7623	0.9038	0.8850	0.8571	0.8941
NB6	0.6483	0.7440	0.8694	0.8188	0.7667	0.8333
NB7	0.6159	0.7143	0.8892	0.8478	0.8485	0.8476
NB17	0.6511	0.7376	0.8688	0.8482	0.6364	0.9367
NB5	0.7243	0.7764	0.8963	0.8188	0.7143	0.8646
NB13	0.7369	0.7764	0.9272	0.8696	0.6981	0.9765
NB14	0.6700	0.7384	0.9043	0.8116	0.6304	0.9022
NB1	0.7561	0.7962	0.9338	0.8759	0.7391	0.9451
RH-1	0.7135	0.7753	0.9233	0.8768	0.7805	0.9175
RH-18	0.5856	0.7084	0.8333	0.8305	0.6000	0.8519
MZ2-MEL	0.7172	0.7693	0.8720	0.7609	0.5079	0.9733

Table S1. Performance measures for 686 GDSC cell lines in the GDSC LOOCV problem.

Supplementary Table S2

Drug	Correlation	C-Index	AUC	Accuracy	Sensitivity	Specificity
Erlotinib	0.1226	0.5557	0.4342	0.9651	0.0000	1.0000
Rapamycin	-0.0951	0.4829	0.4933	0.7246	0.0000	1.0000
Sunitinib	0.0608	0.5237	0.5759	0.6550	0.0000	1.0000
PHA-665752	0.1018	0.5276	0.8123	0.9827	0.0000	1.0000
MG-132	0.0732	0.5208	0.5276	0.7185	0.0000	1.0000
Paclitaxel	0.0278	0.5123	0.5246	0.6327	1.0000	0.0000
Cyclopamine	0.0970	0.5398	0.4286	0.9793	0.0000	1.0000
AZ628	0.2444	0.5808	0.6054	0.7566	0.0000	1.0000
Sorafenib	0.1112	0.5287	0.5438	0.8860	0.0000	1.0000
VX-680	0.0640	0.5336	0.5542	0.7135	0.0000	1.0000
Imatinib	0.0901	0.5412	0.5628	0.9711	0.0000	1.0000
NVP-TAE684	0.0907	0.5343	0.5792	0.5942	0.0142	0.9951
PF-02341066	0.0112	0.5129	0.4792	0.9449	0.0000	1.0000
AZD-0530	0.1856	0.5784	0.5667	0.8844	0.0000	1.0000
S-Trityl-L-cysteine	-0.0297	0.5009	0.5135	0.5497	0.9683	0.0327
Z-LLNle-CHO	0.1193	0.5474	0.5507	0.7953	0.0000	1.0000
Dasatinib	0.1577	0.5665	0.5966	0.6608	0.0000	1.0000
GNF-2	0.0692	0.5373	0.5032	0.9795	0.0000	1.0000
CGP-60474	-0.0381	0.4930	0.5296	0.6023	0.9952	0.0000
CGP-082996	-0.0077	0.5088	0.3533	0.9357	0.0000	1.0000
A-770041	0.1395	0.5657	0.6004	0.7434	0.0000	1.0000
WH-4-023	0.1195	0.5628	0.6019	0.7515	0.0000	1.0000
WZ-1-84	0.0629	0.5240	0.5382	0.9386	0.0000	1.0000
BI-2536	0.0365	0.5280	0.5103	0.5773	0.9949	0.0069
BMS-536924	0.0619	0.5315	0.5645	0.5889	0.1955	0.8381
BMS-509744	0.0136	0.5085	0.5176	0.7959	0.0000	1.0000
CMKd	0.0402	0.5044	0.5306	0.8860	0.0000	0.9967
Pyrimethamine	-0.0817	0.4850	0.4996	0.6804	0.0000	0.9831
JW-7-52-1	0.0205	0.5109	0.5050	0.7638	1.0000	0.0000
A-443654	0.0237	0.5175	0.5484	0.6959	1.0000	0.0000
GW843682X	-0.0033	0.5124	0.4987	0.6764	1.0000	0.0000
MS-275	-0.0695	0.4849	0.4929	0.6765	0.9957	0.0000
Parthenolide	-0.0102	0.5100	0.4146	0.9647	0.0000	1.0000
TGX221	0.1288	0.5488	0.5817	0.9501	0.0000	1.0000
Bortezomib	-0.0406	0.4744	0.4650	0.7118	1.0000	0.0101

XMD8-85	0.0467	0.5169	0.5144	0.7663	0.0000	1.0000
Roscovitine	0.0683	0.5271	0.5514	0.9318	0.0000	1.0000
Salubrinal	-0.0182	0.4806	0.4971	0.8319	0.0000	1.0000
Lapatinib	0.0403	0.5309	0.4430	0.9525	0.0000	1.0000
GSK269962A	0.1291	0.5415	0.6060	0.8651	0.0000	1.0000
Doxorubicin	-0.0159	0.5078	0.4744	0.8428	1.0000	0.0000
Etoposide	0.0106	0.5035	0.5299	0.6845	1.0000	0.0000
Gemcitabine	-0.0489	0.4886	0.5088	0.7396	1.0000	0.0000
Mitomycin C	0.0030	0.5045	0.4373	0.9784	1.0000	0.0000
Vinorelbine	0.0433	0.5223	0.5700	0.7088	0.9893	0.0053
NSC-87877	-0.0294	0.4985	0.4774	0.9848	0.0000	1.0000
Bicalutamide	0.0188	0.5088	0.7886	0.9985	0.0000	1.0000
QS11	-0.0301	0.4947	0.5065	0.7596	0.0065	0.9939
Midostaurin	0.0824	0.5335	0.5454	0.7470	0.0061	0.9939
CHIR-99021	0.0400	0.5212	0.4321	0.9771	0.0000	1.0000
AP-24534	0.0810	0.5301	0.5411	0.8369	0.0000	1.0000
AZD6482d1	0.0301	0.5208	0.5304	0.8201	0.0000	0.9963
JNK-9L	0.0199	0.5123	0.5037	0.9024	1.0000	0.0000
PF-562271	-0.0031	0.4987	0.5070	0.8979	0.0000	1.0000
DMOG	-0.0534	0.4865	0.4767	0.8323	1.0000	0.0000
FTI-277	0.0930	0.5401	0.1363	0.9954	0.0000	1.0000
OSU-03012	0.0059	0.4984	0.4840	0.5208	0.9640	0.0538
Shikonin	-0.0805	0.4774	0.3982	0.8857	1.0000	0.0000
AKT inhibitor VIII	-0.0293	0.4980	0.4750	0.9314	0.0000	1.0000
Embelin	0.0321	0.5172	0.5306	0.6733	1.0000	0.0047
FH535	0.0436	0.5340	0.5173	0.8243	1.0000	0.0000
PAC-1	-0.0815	0.4845	0.4407	0.8891	0.0000	1.0000
IPA-3	0.0244	0.5108	0.5381	0.7827	0.0145	0.9902
GSK-650394	-0.0852	0.4736	0.4596	0.7180	0.0226	0.9788
BAY 61-3606	-0.0442	0.4886	0.4859	0.4946	0.4411	0.5398
Thapsigargin	0.0735	0.5339	0.5382	0.8629	1.0000	0.0000
Obatoclax Mesylate	0.0732	0.5206	0.3744	0.9476	1.0000	0.0000
BMS-754807	0.1108	0.5357	0.5462	0.5470	0.1672	0.9096
OSI-906	0.1009	0.5344	0.5744	0.8706	0.0000	0.9982
Bexarotene	0.0366	0.4971	0.5463	0.9615	0.0000	1.0000
Bleomycin	0.0302	0.5147	0.5241	0.7735	1.0000	0.0000
AUY922	-0.0236	0.4901	0.4988	0.7781	1.0000	0.0000
Bryostatin 1	0.0495	0.5198	0.7106	0.9877	0.0000	1.0000
Pazopanib	0.0558	0.5231	0.5125	0.8860	0.0000	1.0000
LAQ824	0.0100	0.5092	0.4568	0.9569	1.0000	0.0000
Epothilone B	-0.0267	0.4922	0.4848	0.7535	0.9959	0.0000

GSK-1904529A	-0.0428	0.4997	0.2535	0.9969	0.0000	1.0000
Tipifarnib	0.0729	0.5310	0.5391	0.6441	0.9976	0.0213
AS601245	0.0502	0.5143	0.5089	0.7196	0.0111	0.9915
AICAR	-0.1168	0.4621	0.4510	0.4498	0.6769	0.3015
Camptothecin	-0.1000	0.4715	0.4421	0.7796	0.9942	0.0000
Vinblastine	0.0040	0.5023	0.5121	0.8480	0.9982	0.0000
Cisplatin	0.1027	0.5299	0.5403	0.7310	0.0000	0.9959
Cytarabine	0.0999	0.5267	0.5287	0.5471	0.7701	0.2761
Docetaxel	-0.0381	0.4884	0.4550	0.5821	0.8624	0.1275
Methotrexate	-0.0018	0.4978	0.5141	0.7888	0.0075	0.9867
ATRA	0.0604	0.5133	0.5371	0.9483	0.0000	1.0000
Gefitinib	0.0087	0.4981	0.5064	0.9590	0.0000	1.0000
ABT-263	0.0638	0.5175	0.5495	0.7485	0.0122	0.9939
Vorinostat	0.0144	0.5160	0.5507	0.8556	1.0000	0.0000
Nilotinib	0.0273	0.5065	0.5133	0.9719	0.0000	1.0000
RDEA119	0.2315	0.5731	0.5998	0.6271	0.0245	0.9926
CI-1040	0.2442	0.5810	0.6316	0.6330	0.0482	0.9926
Temsirolimus	0.0933	0.5318	0.5483	0.5881	0.1533	0.8741
AZD-2281	0.0292	0.5044	0.5626	0.9711	0.0000	1.0000
ABT-888	-0.0332	0.5010	0.2651	0.9954	0.0000	1.0000
Bosutinib	-0.0074	0.5001	0.5360	0.8328	0.0000	1.0000
Lenalidomide	-0.0339	0.4949	0.3112	0.9939	0.0000	1.0000
Axitinib	0.0842	0.5351	0.5825	0.9210	0.0000	1.0000
AZD7762	-0.0091	0.5043	0.5186	0.7538	1.0000	0.0061
GW 441756	0.0138	0.4987	0.5691	0.9742	0.0000	1.0000
CEP-701	0.0376	0.5134	0.5056	0.7295	0.9855	0.0229
SB 216763	0.0772	0.5204	0.5593	0.9873	0.0000	1.0000
17-AAG	0.1889	0.5595	0.5967	0.5838	0.5610	0.6162
VX-702	-0.0142	0.5087	0.3777	0.9878	0.0000	1.0000
AMG-706	0.0762	0.5346	0.5225	0.9832	0.0000	1.0000
KU-55933	-0.0523	0.4821	0.3400	0.9709	0.0000	1.0000
Elesclomol	-0.0480	0.4881	0.4619	0.7538	0.9940	0.0000
BIBW2992	-0.0356	0.4879	0.4595	0.9119	0.0000	1.0000
GDC-0449	-0.0092	0.5017	0.5401	0.9863	0.0000	1.0000
PLX4720	0.1194	0.5303	0.6639	0.9131	0.0000	1.0000
BX-795	0.0595	0.5277	0.5441	0.5933	0.0775	0.9293
NU-7441	-0.0126	0.4967	0.4584	0.9709	0.0000	1.0000
SL 0101-1	0.0194	0.5172	0.1951	0.9985	0.0000	1.0000
BIRB 0796	0.0329	0.5081	0.5221	0.9832	0.0000	1.0000
JNK Inhibitor VIII	0.0358	0.5101	0.9985	0.9970	0.0000	0.9985
681640	-0.0120	0.4984	0.5467	0.9167	0.0000	1.0000

Nutlin-3a	0.3678	0.6030	0.7497	0.8308	0.0000	0.9982
PD-173074	0.0286	0.5132	0.5078	0.9665	0.0000	1.0000
ZM-447439	-0.0103	0.4956	0.4681	0.8116	0.0083	1.0000
RO-3306	0.0801	0.5144	0.6296	0.9512	0.0000	1.0000
MK-2206	0.0016	0.5027	0.4853	0.7150	0.0286	0.9801
PD-0332991	0.0539	0.5218	0.5379	0.7134	0.0166	0.9955
NVP-BEZ235	0.0029	0.4989	0.4945	0.7997	1.0000	0.0000
GDC0941	-0.0361	0.4829	0.4840	0.4760	0.4094	0.5256
AZD8055	0.0406	0.5073	0.5154	0.7103	1.0000	0.0053
PD-0325901	0.2074	0.5697	0.6003	0.7304	0.0057	0.9979
SB590885	0.0257	0.5057	0.5714	0.9481	0.0000	1.0000
AZD6244	0.0541	0.5283	0.5666	0.8455	0.0000	1.0000
AZD6482d2	0.0308	0.5197	0.5090	0.8210	0.0000	1.0000
CCT007093	0.0340	0.5100	0.4154	0.9939	0.0000	1.0000
EHT 1864	-0.0352	0.4946	0.5329	0.9647	0.0000	1.0000
BMS-708163	0.0019	0.4987	0.4119	0.9908	0.0000	1.0000
PF-4708671	-0.0306	0.4892	0.4576	0.9861	0.0000	1.0000
JNJ-26854165	0.0479	0.5172	0.5270	0.7273	0.0114	0.9916
TW 37	0.0327	0.5027	0.4518	0.9353	1.0000	0.0000
CCT018159	-0.0859	0.4677	0.3952	0.7203	0.0000	0.9584
AG-014699	0.1367	0.5385	0.6240	0.9615	0.0000	1.0000

Table S2. Performance measures for the 138 GDSC drugs in the GDSC LOOCV problem.

Supplementary Table S3

Cell Line	Correlation	C-Index	AUC	Accuracy	Sensitivity	Specificity
22Rv1	0.6857	0.6545	1.0000	1.0000	1.0000	1.0000
42-MG-BA	0.7918	0.8056	1.0000	1.0000	1.0000	1.0000
786-O	0.6941	0.7333	1.0000	1.0000	1.0000	1.0000
A-204	0.2881	0.6727	0.7143	0.8182	0.5000	1.0000
A-253	0.7262	0.6545	1.0000	1.0000	1.0000	1.0000
A-375	0.7979	0.8000	0.9333	0.8182	0.6000	1.0000
A-673	0.5032	0.7333	1.0000	0.7000	1.0000	0.6250
ALL-SIL	0.1300	0.4909	0.8333	0.9091	0.6667	1.0000
AMO-1	0.2878	0.6364	0.8929	0.8182	0.5000	1.0000
AN3 CA	0.7322	0.7455	1.0000	1.0000	1.0000	1.0000
B-CPAP	0.4799	0.6727	1.0000	1.0000	1.0000	1.0000
BDCM	0.7555	0.9455	0.9667	0.8182	0.6000	1.0000
CAL 27	0.3080	0.6444	0.6250	0.6000	0.3333	1.0000

CAL-78	0.7010	0.6545	1.0000	1.0000	1.0000	1.0000
CCK-81	0.3774	0.5833	0.7000	0.6667	0.4000	1.0000
CI-1	0.7370	0.7091	1.0000	0.9091	0.6667	1.0000
COLO 201	0.9643	0.8182	1.0000	0.9091	0.8333	1.0000
COLO 205	0.8922	0.8545	1.0000	1.0000	1.0000	1.0000
COLO 741	0.9577	0.8889	1.0000	1.0000	1.0000	1.0000
COLO-320	0.5154	0.5818	0.9444	0.9091	1.0000	0.8889
COV318	-0.4499	0.4000	0.0000	0.0000	0.0000	0.0000
COV504	0.7597	0.7273	1.0000	1.0000	1.0000	1.0000
Caki-2	0.6757	0.6364	1.0000	1.0000	1.0000	1.0000
Calu-1	0.6091	0.6545	0.9444	0.9091	1.0000	0.8889
DMS 114	0.6686	0.6182	1.0000	1.0000	1.0000	1.0000
DU 145	0.5672	0.5636	1.0000	1.0000	1.0000	1.0000
DV-90	0.8960	0.8889	1.0000	0.7778	0.5000	1.0000
Detroit 562	0.7708	0.6909	1.0000	1.0000	1.0000	1.0000
EBC-1	0.5549	0.8000	0.8000	0.7273	0.4000	1.0000
EFE-184	0.4172	0.6727	0.9000	0.9091	1.0000	0.9000
EN	0.5228	0.5273	1.0000	1.0000	1.0000	1.0000
ES-2	0.7054	0.7333	0.8750	0.8000	0.5000	1.0000
F-36P	0.5691	0.7273	0.8667	0.7273	0.4000	1.0000
FU-OV-1	0.7353	0.7455	1.0000	1.0000	1.0000	1.0000
FU97	0.9068	0.8889	1.0000	0.8889	0.6667	1.0000
FaDu	0.3483	0.6182	0.6429	0.8182	0.5000	1.0000
GRANTA-519	0.2270	0.5636	0.6667	0.9091	0.6667	1.0000
HARA	0.8012	0.8056	1.0000	1.0000	1.0000	1.0000
HCC-15	0.6558	0.6727	1.0000	1.0000	1.0000	1.0000
HCC-44	0.8683	0.7778	1.0000	1.0000	1.0000	1.0000
HCC-56	0.7864	0.7778	0.9524	0.8000	0.3333	1.0000
HCC-78	0.6239	0.6727	1.0000	0.9091	0.5000	1.0000
HCC2935	-0.1366	0.4364	0.3750	0.7273	0.3333	0.8750
HCC4006	0.2361	0.5636	0.5714	0.8182	0.5000	1.0000
HCC827	0.2049	0.5278	0.6000	0.7778	0.5000	1.0000
HCT 116	0.7861	0.8545	0.9643	0.8182	0.5000	1.0000
HEC-1-A	0.6190	0.6000	1.0000	1.0000	1.0000	1.0000
HEC-1-B	0.4109	0.6727	0.8333	0.9091	0.6667	1.0000
HEC-151	0.7856	0.7636	0.9643	0.8182	0.5000	1.0000
HEC-251	0.6952	0.6727	1.0000	1.0000	1.0000	1.0000
HEC-265	0.6807	0.7091	1.0000	1.0000	1.0000	1.0000
HEC-59	0.5652	0.6727	1.0000	1.0000	1.0000	1.0000
HEC-6	0.3676	0.5818	1.0000	1.0000	1.0000	1.0000
HEL 92.1.7	0.6921	0.6909	1.0000	1.0000	1.0000	1.0000

HLF	0.6878	0.6545	1.0000	1.0000	1.0000	1.0000
HMC-1-8	0.7839	0.7818	0.9286	0.8182	0.5000	1.0000
HMCB	0.4431	0.5273	1.0000	1.0000	1.0000	1.0000
HUP-T3	0.8529	0.8364	0.9643	0.8182	0.5000	1.0000
HUP-T4	0.6423	0.6909	0.9643	0.8182	0.5000	1.0000
Hep G2	0.8110	0.8727	0.9643	0.9091	0.7500	1.0000
Hey-A8	0.7358	0.8222	1.0000	0.9000	1.0000	0.8571
Hs 229.T	0.6480	0.6909	1.0000	1.0000	1.0000	1.0000
Hs 578T	0.6808	0.7091	1.0000	1.0000	1.0000	1.0000
Hs 683	0.7013	0.7091	1.0000	0.9091	1.0000	0.8889
Hs 695T	0.6707	0.7818	1.0000	1.0000	1.0000	1.0000
Hs 729	0.8010	0.6389	1.0000	1.0000	1.0000	1.0000
Hs 739.T	0.6952	0.6909	1.0000	1.0000	1.0000	1.0000
Hs 746T	0.7277	0.7273	1.0000	1.0000	1.0000	1.0000
Hs 766T	0.6081	0.7455	0.8333	0.8182	0.6667	0.8750
Hs 840.T	0.6180	0.7091	1.0000	1.0000	1.0000	1.0000
Hs 852.T	0.6699	0.7455	1.0000	1.0000	1.0000	1.0000
Hs 895.T	0.4094	0.6000	0.8333	0.7273	0.3333	0.8750
Hs 936.T	0.7469	0.8545	0.9643	0.8182	0.7500	0.8571
Hs 944.T	0.8351	0.8727	0.9643	0.8182	0.5000	1.0000
HuT 78	0.5356	0.7636	0.8000	0.7273	0.4000	1.0000
IGR-37	0.6271	0.6727	0.8214	0.6364	0.2500	0.8571
IGR-39	0.4953	0.5818	1.0000	0.9091	1.0000	0.9000
IGROV1	0.6833	0.6667	1.0000	1.0000	1.0000	1.0000
IM95	0.8065	0.8000	0.9643	0.8182	0.5000	1.0000
IMR-32	0.6780	0.7455	1.0000	1.0000	1.0000	1.0000
IST-MES2	0.5961	0.6909	0.9583	0.9091	0.6667	1.0000
Ishikawa (Heraklio) 02 ER-	0.7057	0.6944	1.0000	1.0000	1.0000	1.0000
JHH-2	0.4702	0.5818	0.9000	0.8182	1.0000	0.8000
JHH-4	0.5497	0.6000	1.0000	0.9091	1.0000	0.9000
JHH-5	0.5668	0.8611	0.7778	0.5556	0.3333	1.0000
JHH-6	0.5970	0.7091	1.0000	0.9091	1.0000	0.8889
JHH-7	0.8786	0.9167	1.0000	0.8889	0.6667	1.0000
JHOS-2	0.6738	0.6909	1.0000	1.0000	1.0000	1.0000
JHOS-4	0.4725	0.5455	1.0000	1.0000	1.0000	1.0000
JHUEM-2	0.8294	0.8056	1.0000	1.0000	1.0000	1.0000
JM1	0.5451	0.6545	1.0000	0.9091	1.0000	0.8889
JMSU-1	0.5201	0.6364	1.0000	0.9091	0.6667	1.0000
K029AX	0.8375	0.8182	0.9643	0.9091	0.7500	1.0000
KARPAS-620	0.8503	0.8182	1.0000	0.7273	0.4000	1.0000
KASUMI-2	0.7049	0.7091	1.0000	1.0000	1.0000	1.0000

KCL-22	0.5208	0.7091	0.7333	0.7273	0.4000	1.0000
KE-39	0.7011	0.7455	1.0000	1.0000	1.0000	1.0000
KE-97	0.5204	0.6000	1.0000	1.0000	1.0000	1.0000
KELLY	0.7843	0.6667	1.0000	0.8889	1.0000	0.8750
KHM-1B	0.1832	0.5636	1.0000	0.9091	1.0000	0.8889
KMBC-2	0.7726	0.8545	1.0000	0.9091	0.6667	1.0000
KMM-1	0.1959	0.6182	0.7857	0.8182	0.5000	1.0000
KMRC-1	0.6200	0.6909	1.0000	1.0000	1.0000	1.0000
KMRC-2	0.4691	0.6909	0.9000	0.8182	0.0000	0.9000
KMS-11	0.5122	0.6364	1.0000	1.0000	1.0000	1.0000
KMS-12-BM	0.5707	0.6364	1.0000	1.0000	1.0000	1.0000
KMS-26	0.6674	0.6000	0.9583	0.9091	0.6667	1.0000
KMS-34	0.5039	0.6545	1.0000	0.9091	0.6667	1.0000
KNS-60	0.6773	0.7091	1.0000	1.0000	1.0000	1.0000
KO52	0.7858	0.8000	0.9667	0.6364	0.3333	1.0000
KP-1N	0.5969	0.6909	1.0000	0.9091	1.0000	0.8889
KP-1NL	0.5851	0.6909	1.0000	0.9091	1.0000	0.8889
KP-2	0.7942	0.8000	0.9167	0.8000	0.5000	1.0000
KP-3	0.7637	0.7273	1.0000	1.0000	1.0000	1.0000
KP-N-SI9s	0.8659	0.8727	1.0000	0.8182	0.6000	1.0000
KP4	0.4666	0.7091	0.7500	0.9091	0.6667	1.0000
KYM-1	0.7813	0.8056	1.0000	0.8889	0.6667	1.0000
KYSE-30	0.7655	0.7636	0.9286	0.7273	0.2500	1.0000
L3.3	0.4315	0.5818	1.0000	0.9091	1.0000	0.8889
LC-1/sq-SF	0.6286	0.6727	1.0000	1.0000	1.0000	1.0000
LN-18	0.4863	0.6444	1.0000	1.0000	1.0000	1.0000
LN-229	0.7142	0.6727	1.0000	1.0000	1.0000	1.0000
LOU-NH91	0.5434	0.5091	1.0000	0.9091	1.0000	0.9000
LOX IMVI	0.7355	0.8444	0.9583	0.9000	0.7500	1.0000
LS123	0.6040	0.6727	1.0000	0.9091	1.0000	0.8889
LS411N	0.9038	0.9167	1.0000	0.6667	0.4000	1.0000
LS513	0.7305	0.8545	1.0000	1.0000	1.0000	1.0000
LU99	0.9024	0.9091	0.9643	0.9091	0.7500	1.0000
LUDLU-1	0.2798	0.5273	0.7500	0.9091	0.6667	1.0000
MCAS	0.7938	0.8545	1.0000	1.0000	1.0000	1.0000
MDA-MB-435S	0.9264	0.8545	1.0000	0.8182	0.6000	1.0000
MDA-MB-436	0.4286	0.5091	1.0000	0.9091	1.0000	0.9000
MEC-1	0.5878	0.6000	1.0000	1.0000	1.0000	1.0000
MFE-319	0.3913	0.6667	0.6000	0.7778	0.5000	1.0000
MIA PaCa-2	0.8272	0.8000	0.9643	0.8182	0.5000	1.0000
MJ	0.7080	0.7273	1.0000	0.9091	1.0000	0.8889

MKN74	0.6028	0.6545	1.0000	1.0000	1.0000	1.0000
MOG-G-CCM	0.7207	0.6364	1.0000	1.0000	1.0000	1.0000
MOLP-8	0.6530	0.7273	0.9167	0.8182	0.6667	0.8750
MONO-MAC-1	-0.1021	0.4545	0.6429	0.8182	0.5000	1.0000
MOR/CPR	0.7335	0.6944	1.0000	1.0000	1.0000	1.0000
MPP 89	0.6939	0.6364	1.0000	1.0000	1.0000	1.0000
Malme-3M	0.8143	0.7818	1.0000	1.0000	1.0000	1.0000
MeWo	0.4556	0.5091	1.0000	0.9091	1.0000	0.9000
Mino	0.4774	0.6727	1.0000	0.9091	0.6667	1.0000
NCI-H1184	0.7021	0.6727	1.0000	1.0000	1.0000	1.0000
NCI-H1339	0.6354	0.6000	1.0000	1.0000	1.0000	1.0000
NCI-H1341	0.6650	0.6727	1.0000	1.0000	1.0000	1.0000
NCI-H1373	0.4182	0.6727	0.9000	0.9091	1.0000	0.9000
NCI-H1568	0.3296	0.4364	1.0000	0.9091	1.0000	0.9000
NCI-H1869	0.2392	0.5455	0.9000	0.8182	1.0000	0.8000
NCI-H1915	0.7866	0.8727	0.9643	0.7273	0.2500	1.0000
NCI-H1944	0.7940	0.8000	1.0000	0.9091	1.0000	0.8750
NCI-H2023	0.5647	0.6000	1.0000	1.0000	1.0000	1.0000
NCI-H211	0.5467	0.6182	1.0000	1.0000	1.0000	1.0000
NCI-H2172	0.4807	0.7455	0.8214	0.8182	0.5000	1.0000
NCI-H2286	0.6888	0.7636	1.0000	0.8182	0.5000	1.0000
NCI-H2444	0.7005	0.8000	0.8571	0.8182	0.5000	1.0000
NCI-H3255	0.1653	0.4909	0.4583	0.7273	0.3333	0.8750
NCI-H647	0.6805	0.6364	1.0000	1.0000	1.0000	1.0000
NCO2	0.2131	0.6545	0.8000	0.6364	0.3333	1.0000
NIH:OVCAR-3	0.4437	0.6364	1.0000	1.0000	1.0000	1.0000
NUGC-4	0.4080	0.7455	0.8214	0.6364	0.4286	1.0000
OC 316	0.5852	0.6727	1.0000	1.0000	1.0000	1.0000
OCI-AML5	0.7824	0.8545	0.8929	0.8182	0.5000	1.0000
OCI-LY10	0.7947	0.6667	1.0000	1.0000	1.0000	1.0000
OCUM-1	0.8430	0.8545	0.9643	0.9091	0.7500	1.0000
OE21	0.3352	0.6909	0.6786	0.6364	0.4286	1.0000
ONCO-DG-1	0.5927	0.5818	1.0000	1.0000	1.0000	1.0000
OV-90	0.7571	0.7636	0.9286	0.8182	0.5000	1.0000
OVMANA	0.4408	0.6364	0.9000	0.9091	1.0000	0.9000
OVSAHO	0.4174	0.6182	0.0000	0.0000	0.0000	0.0000
OVTOKO	0.3564	0.6000	0.7778	0.8000	1.0000	0.7778
P31/FUJ	0.7751	0.8364	0.9643	0.8182	0.5000	1.0000
P3HR-1	0.6767	0.7455	1.0000	1.0000	1.0000	1.0000
PA-TU-8902	0.3848	0.6389	0.8750	0.8889	1.0000	0.8750
PK-1	0.7229	0.7455	1.0000	0.9091	0.6667	1.0000

PK-45H	0.8066	0.8545	1.0000	0.9091	0.7500	1.0000
PK-59	0.7670	0.7636	1.0000	0.9091	0.6667	1.0000
Panc 02.03	0.6967	0.7636	0.9643	0.9091	0.7500	1.0000
Panc 03.27	0.7083	0.7273	0.9444	0.9091	1.0000	0.8889
Panc 04.03	0.6309	0.7273	0.8929	0.8182	0.5000	1.0000
Panc 10.05	0.6729	0.7091	0.9583	0.9091	0.6667	1.0000
Pfeiffer	0.7520	0.6727	1.0000	1.0000	1.0000	1.0000
QGP-1	0.9096	0.8611	1.0000	0.8889	0.7500	1.0000
RERF-GC-1B	0.7486	0.6364	1.0000	1.0000	1.0000	1.0000
RERF-LC-AI	0.6832	0.7091	1.0000	1.0000	1.0000	1.0000
Reh	0.7167	0.7636	1.0000	0.9091	0.5000	1.0000
SCaBER	0.2607	0.6000	0.6333	0.6364	0.2000	1.0000
SF-295	0.7273	0.6364	1.0000	1.0000	1.0000	1.0000
SH-10-TC	0.6792	0.6364	1.0000	1.0000	1.0000	1.0000
SK-BR-3	0.5953	0.6545	0.8333	0.9091	0.6667	1.0000
SK-ES-1	0.6706	0.7091	1.0000	1.0000	1.0000	1.0000
SK-N-SH	0.4049	0.5636	0.8889	0.9091	1.0000	0.8889
SNU-1	0.8127	0.8727	1.0000	0.9091	0.6667	1.0000
SNU-182	0.6716	0.7273	1.0000	1.0000	1.0000	1.0000
SNU-398	0.7425	0.8056	1.0000	0.8889	0.6667	1.0000
SNU-C2A	0.6953	0.6545	1.0000	1.0000	1.0000	1.0000
SU-DHL-10	0.6694	0.7455	0.8929	0.8182	0.5000	1.0000
SU-DHL-4	0.4812	0.7273	1.0000	0.8182	0.3333	1.0000
SU-DHL-6	0.1999	0.5636	0.6429	0.8182	0.5000	1.0000
SU-DHL-8	0.4871	0.6545	0.9583	0.9091	0.6667	1.0000
SU.86.86	0.6184	0.6667	1.0000	0.9000	1.0000	0.8750
SUP-M2	0.8167	0.8000	0.9583	0.9091	0.6667	1.0000
SW 1088	0.4498	0.5818	0.7083	0.9091	0.6667	1.0000
SW 1271	0.8160	0.8727	1.0000	0.8182	0.5000	1.0000
SW 1353	0.6472	0.6545	1.0000	1.0000	1.0000	1.0000
SW 1573	0.5233	0.5278	1.0000	0.8889	1.0000	0.8750
SW 1990	0.7707	0.8000	1.0000	0.9091	1.0000	0.8750
SW 780	0.8852	0.9091	1.0000	0.8182	0.5000	1.0000
SW 900	0.7479	0.6909	1.0000	1.0000	1.0000	1.0000
SW403	0.7201	0.8000	0.9286	0.8182	0.5000	1.0000
SW480	0.8110	0.8727	1.0000	1.0000	1.0000	1.0000
SW579	0.6348	0.6545	1.0000	1.0000	1.0000	1.0000
Sq-1	0.7744	0.7222	1.0000	1.0000	1.0000	1.0000
T-47D	0.3611	0.6364	0.9000	0.9091	1.0000	0.9000
T3M-10	0.7733	0.7091	1.0000	0.9091	0.6667	1.0000
TC-71	0.7037	0.6909	1.0000	0.9091	0.5000	1.0000

TE 617.T	0.3885	0.7455	0.7857	0.5455	0.2857	1.0000
TEN	0.7173	0.6727	1.0000	1.0000	1.0000	1.0000
TOV-21G	0.6382	0.5818	1.0000	1.0000	1.0000	1.0000
TT2609-C02	0.8512	0.8364	0.9286	0.8182	0.5000	1.0000
Toledo	0.7565	0.7091	1.0000	0.9091	1.0000	0.9000
U-2 OS	0.6491	0.6545	1.0000	1.0000	1.0000	1.0000
U-87 MG	0.6683	0.7455	1.0000	1.0000	1.0000	1.0000
U-937	0.8293	0.7818	1.0000	0.9091	0.6667	1.0000
VMRC-LCD	0.7025	0.7273	0.9583	0.9091	0.6667	1.0000
VMRC-RCW	-0.5506	0.2909	0.2000	0.7273	0.0000	0.8000
WM-266-4	0.9743	0.8889	1.0000	1.0000	1.0000	1.0000
WM-793	0.6487	0.7273	1.0000	1.0000	1.0000	1.0000
WM-88	0.7915	0.8182	0.9333	0.7273	0.4000	1.0000
WM-983B	0.9354	0.8545	1.0000	0.6364	0.3333	1.0000
WM1799	0.8491	0.8000	1.0000	0.9091	0.8000	1.0000
YKG1	0.6990	0.7091	1.0000	1.0000	1.0000	1.0000
ZR-75-1	0.5845	0.7500	1.0000	1.0000	1.0000	1.0000

Table S3. Performance measures for 235 CCLE-only cell lines in the CCLE validation problem.

Supplementary Table S4

Drug	Correlation	C-Index	AUC	Accuracy	Sensitivity	Specificity
17-AAG	-0.0274	0.5007	0.4573	0.0894	0.9286	0.0362
AZD6244	0.0617	0.5514	0.5191	0.9489	0.0000	0.9696
Erlotinib	-0.1782	0.4813	0.1978	0.0596	0.0000	1.0000
Lapatinib	-0.0895	0.4751	0.4097	0.9660	0.0000	1.0000
Nilotinib	0.0152	0.5019	0.5305	0.9234	0.0000	1.0000
PD-0325901	0.1059	0.5339	0.5954	0.7647	0.3333	0.7846
PD-0332991	0.0882	0.5229	0.6058	0.0812	0.0138	1.0000
PHA-665752	0.0170	0.5456	0.5242	0.9520	0.0000	1.0000
PLX4720	0.0913	0.5433	0.5928	0.8565	0.0000	0.9676
Paclitaxel	-0.1228	0.4520	0.4632	0.3745	1.0000	0.0068
Sorafenib	0.0161	0.5251	0.4888	0.7660	0.0000	1.0000

Table S4. Performance measures for 11 shared GDSC and CCLE drugs in the CCLE validation problem.
